# Efficacy and safety of lenvatinib plus transarterial chemoembolization with or without programmed death-1 inhibitors in the treatment of intermediate or advanced hepatocellular carcinoma: a systematic review and meta-analysis

**DOI:** 10.3389/fimmu.2025.1586914

**Published:** 2025-07-24

**Authors:** Yongfa Lei, Xiaotian Liang, Hua Zhu, Jin Wang, Xiaochen Zhang, Siliang Duan, Weiming Liang

**Affiliations:** ^1^ The First Affiliated Hospital of Guangxi University of Science and Technology, Guangxi University of Science and Technology, Liuzhou, Guangxi, China; ^2^ Medicine College, Guangxi University of Science and Technology, Liuzhou, Guangxi, China

**Keywords:** hepatocellular carcinoma, lenvatinib, PD-1 inhibitor, transarterial chemoembolization, progression-free survival, overall survival, objective response rate, meta-analysis

## Abstract

**Introduction:**

This meta-analysis aimed to evaluate the efficacy and safety of Lenvatinib plus transarterial chemoembolization with or without programmed death-1 inhibitors (PD-1 inhibitors) in the treatment of intermediate or advanced hepatocellular carcinoma (HCC).

**Materials and Methods:**

Four databases (Pubmed, Embase, Web of Science, and Cochrane Library) were searched for studies comparing lenvatinib plus transarterial chemoembolization with PD-1 inhibitors (TACE-L-P) versus Lenvatinib plus transarterial chemoembolization (TACE-L) for intermediate or advanced HCC. Meta-analyses were conducted for progression-free survival (PFS), overall survival (OS), objective response rate (ORR), disease control rate (DCR), and Grade ≥ 3 treatment-related adverse events (Grade ≥ 3 AEs).

**Results:**

The meta-analysis comprised 19 retrospective cohort studies, including of 2002 patients diagnosed with intermediate or advanced HCC. In this cohort, 1011 individuals were administered TACE-L-P, while 991 patients received TACE-L. In comparison to TACE-L, TACE-L-P demonstrated a superior ORR [odds ratio (OR) = 2.38, 95% confidence interval (CI) 1.98 ~ 2.87, P < 0.00001] and DCR (OR = 3.22, 95% CI, 2.32 ~ 4.45, P < 0.00001). TACE-L-P showed superior efficacy compared to TACE-L regarding PFS (HR: 0.56, 95%CI 0.50 to 0.62, P<0.0001) and OS (HR: 0.70, 95%CI 0.60 to 0.80, P<0.0001). Regarding safety, the incidence of Grade ≥ 3 AEs was more prevalent in the TACE-L-P group compared to the TACE-L group (OR=1.58, 95% CI: 1.27 ~ 1.97, P<0.0001).

**Conclusions:**

The present meta-analysis present a comparison of the efficacy and safety of TACE-L-P against TACE-L for intermediate or advanced HCC. TACE-L-P enhanced ORR, DCR, PFS, and OS relative to TACE-L. Furthermore, the improved efficacy of TACE-L-P was correlated with a rise in the incidence of Grade ≥ 3 AEs.

**Systematic review registration:**

https://www.crd.york.ac.uk/prospero/display_record.php?ID=CRD42024590414, identifier CRD42024590414.

## 1 Introduction

Hepatocellular carcinoma (HCC) is the predominant form of primary liver cancer, representing 80-90% of incidence (1). This malignancy imposes a significant health and economic cost worldwide, particularly in Asia (2). With a 5-year survival rate of around 18%, it ranks as the third most common cause of cancer-related mortality worldwide (3). Possible therapeutic interventions encompass surgical excision, image-guided ablation, liver transplantation, transarterial chemoembolization (TACE), sorafenib, and lenvatinib. Specific therapeutic interventions are recommended during certain clinical phases, and it is common to employ combination therapies (4–6). While surgical resection, ablation, and liver transplantation have the potential to completely cure HCC, most patients are found with advanced disease that is not responsive to these treatments. As a result, their prognosis is dismal, with an anticipated median life of 6–8 months (7–9).

The REFLECT trial validated those patients who received lenvatinib treatment had extended progression-free survival (PFS) and improved objective response rate (ORR). The findings corroborated those of prior investigations on sorafenib, leading to the recognition of lenvatinib as a novel therapy for advanced-stage HCC (10). Lenvatinib is an orally delivered, multi-targeted tyrosine kinase inhibitor that precisely blocks vascular endothelial growth factor receptor, fibroblast growth factor receptor (FGFR) 1–4, platelet-derived growth factor receptor- α (PDGFR α) and receptor tyrosine kinase (KIT) (11, 12). Lenvatinib has been shown in preclinical investigations to effectively inhibit vascular endothelial growth factor (VEGF) and fibroblast growth factor (FGF) induced angiogenesis and VEGFR3-associated lymphangiogenesis (12–15). Furthermore, TACE has demonstrated the capacity to be used with immunotherapy (16). The anticancer mechanism of TACE entails the lowering of tumor size via the obstruction of blood flow to acquire a therapeutic outcome (17, 18). Considering the high incidence of tumor recurrence following TACE, this operation is often performed multiple times. Nevertheless, the repeated administration of TACE may cause failure of liver function, leading to a negative prognosis for the patient (19). Moreover, TACE induces tumor hypoxia, resulting in the increased expression of hypoxia inducible factor-1α (HIF-1α). Elevated levels of HIF-1α subsequently stimulate the promotion of vascular endothelial growth factor and platelet-derived growth factor (PDGF) production, hence enhancing tumor angiogenesis (20–22). Compared to TACE monotherapy, a previous study indicated that combination therapy with lenvatinib and TACE had a better tendency for extending the survival of patients with unresectable HCC (23).

Immune checkpoint inhibitors (ICIs) immunotherapies enhance the immune tresponses against cancer by specifically targeting immunologic receptors located on the surface of T-lymphocytes or cancer cells (24). T cells express programmed death-1 inhibitors (PD-1 inhibitors) and cytotoxic T lymphocyte antigen 4 as co-inhibitory receptors on their surface to suppress T cell-mediated immune responses. However, cancer cells manipulate these inhibitory molecules to promote tumor tolerance and T cell exhaustion (25). More recently, immune checkpoint inhibitors, such as PD-1 and programmed death ligand 1 (PD-L1) inhibitors, have shown a potential therapeutic advantage for patients with advanced HCC (26). Recent studies indicated that utilizing triple therapy of TACE, Lenvatinib, and PD-1/L1 inhibitor might enhance the combined anti-cancer effects in advanced HCC and lead to better efficacy (27, 28). Yan et al. conducted a multicenter retrospective study involving 62 patients with unresectable HCC who received lenvatinib and PD-1 inhibitors in conjunction with TACE; the overall response rate (ORR) was 77.4%, and 53.2% of patients were converted to resectable HCC (29). Other studies also reported that lenvatinib plus transarterial chemoembolization with PD-1 inhibitors (TACE-L-P) provided better ORR and PFS than Lenvatinib plus transarterial chemoembolization (TACE-L) for patients with unresectable HCC (30, 31). The above results have all achieved positive results, indicating that PD-1 inhibitors can improve the prognosis of patients with TACE and lenvatinib. Regarding safety, the treatment-related adverse events (AEs) were controllable and acceptable in both groups. The incidence of grade 3–4 adverse events was greater in the TACE-L-P group compared to the TACE-L group, which remained safe with suitable symptomatic management (32). The primary Grade≥ 3 adverse events reported included abdominal pain, decreased appetite, diarrhea, fever, fatigue, hand-foot syndrome, hypertension, nausea, and rashes, among others. These adverse responses primarily pertain to the therapeutic mechanism of PD-1 immune checkpoint inhibition. The combination therapy for intermediate or advanced hepatocellular carcinoma can result in unavoidable adverse responses, necessitating continuous scrutiny of its long-term safety (32, 33).

Nevertheless, the effectiveness and safety of TACE-L-P in intermediate or advanced HCC remains somewhat contentious. Given the growing use of TACE-L-P, understanding their efficacy and safety profile is critical. Hence, we conducted this meta-analysis with the aim of providing clearer insights into the effectiveness and safety of TACE-L-P versus TACE-L for intermediate or advanced HCC and informing clinical decision-making.

## 2 Materials and methods

### 2.1 Search strategy

The current meta-analysis was conducted in compliance with the 2020 guidelines of the Preferred Reporting Project for Systematic Review and Meta-Analysis (PRISMA) (33) and the Assessing the methodological quality of systematic reviews (AMSTAR). A comprehensive literature search was conducted across four electronic databases, including of PubMed, Embase, the Cochrane Library and Web of Science, to identify pertinent articles published in the period from their inception to May 16, 2025. The search keywords were: “hepatocellular carcinoma” AND “Lenvatinib” AND “transarterial chemoembolization” AND “PD-1 inhibitor” AND “study”. Supplementary Tables provides a comprehensive listing of the search results. A comprehensive manual review of the bibliographies of the identified papers, together with relevant reviews and meta-analyses, was undertaken to identify any new research that satisfied the inclusion criteria. Furthermore, we performed a comprehensive search on three clinical trial registries, specifically ClinicalTrials.gov, Controlled-trials.com, and Umin.ac.jp/ctr/index.htm, to ensure the incorporation of unpublished data.

### 2.2 Inclusion and exclusion criteria

The criteria for inclusion were as follows: (1) patients: diagnosed with intermediate or advanced HCC [Barcelona Clinic Liver Cancer (BCLC) Stage B or C], Eastern Cooperative Oncologic Group (ECOG) score of 0 to 1, Child-Pugh class A/B. Intermediate HCC, or BCLC Stage B HCC, included asymptomatic patients with multinodular tumors without vascular invasion or extrahepatic spread (34, 35). Advanced HCC, or BCLC Stage C HCC, comprised patients with either symptomatic tumors or with an invasive tumoral pattern reflected by the presence of vascular invasion or extrahepatic spread (34, 35). (2) patient in the intervention group received TACE-L-P. (3) patient in the control group received TACE-L. (4) at least one of the following outcomes were reported: ORR, Disease control rate (DCR), Overall survival (OS), PFS, Grade≥ 3AEs. (5) study types: randomized controlled trials, prospective studies, or retrospective studies.

The criteria for exclusion were as follows: (1) other types of articles, including case reports, publications, letters, reviews, meta-analyses, editorials, animal studies, and protocols; (2) other types of cancers; (3) absence of relative outcomes; (4) duplicate group of patients; and (5) inability to compile data for meta-analysis.

### 2.3 Selection of studies

Selection of studies, including elimination of duplicates, was undertaken using EndNote (Version 20; Clarivate Analytics). An initial search was undertaken by two reviewers who independently deleted duplicate entries, assessed the titles and abstracts for relevance, and classified each study as either included or excluded. The settlement was arrived at through the attainment of consensus. A third author of the review would take on the role of an arbitrator if lacking a consensus.

### 2.4 Data extraction

Two separate reviewers conducted a thorough examination of the title and abstract, followed by a comprehensive review of the entire text. In order to resolve the discrepancies, expert advice was sought from a third investigator. The collected data comprises the first author’s name, publication year, study area, trial ID, study design, sample size, intervention, participant age, trial phase, study design, sample size, study period, median follow-up duration, ORR, DCR, Kaplan-Meier (KM) curves for PFS, KM curves for OS, and Grade ≥3 AEs. If the same cohort of patients were reported in several publications, only the latest data would be retained to avoid the duplication of information.

### 2.5 Quality assessment

The revised Newcastle-Ottawa Scale (NOS) developed by Lo, Mertz, and Loeb in 2014 was employed to assess the quality of the included studies (36). Two reviewers independently evaluated each study from three domains: (1) Selection of the cohort (4 items), including of representativeness of the case/exposure group (1 point), selection of the non-case/non-exposure group (1 point), definition of exposure (1 point) and no relevant outcome at the start of the study (1 point); (2) Comparability (2 items), including of comparability on most important factors (up to 2 points) and comparability on other risk factors (1 point); (3) Outcome determination (3 items), including of outcome assessment (1 point), dequacy of follow-up time (1 point) and follow-up completeness (1 point). Studies with scores of ≥7 were classified as high quality. In case of any discrepancy, a consensus was formed by mutual discussion with other reviewers.

### 2.6 Evidence certainty

The certainty of evidence for the systematic review was assessed by two independent reviewers using the GRADEpro GDT (37): GRADEpro Guideline Development Tool [Software]. McMaster University and Evidence Prime, 2021. Available from gradepro.org. In case of any discrepancy, a consensus was formed by mutual discussion with other reviewers.

### 2.7 Statistical analysis

The data from papers providing Kaplan-Meier curves was extracted using GraphPad Prism software. The individual data were then recreated using the IPDformKM utility. The proven methodology developed by Guyot et al. was employed to recreate data at the level of individual patients (38). The procedure was conducted in a user-friendly Shiny application developed by Guyot et al, which is freely available at https://www.trialdesign.org/one-page-shell.html#IPDfromKM. Quantitative analysis was performed using Review Manager v5.3 software. The choice between fixed - effect and random-effect models was based on the I² value and chi-square test P value. When heterogeneity was high (I² >50%), the random-effect model was used. When heterogeneity was low (I² ≤50%), the fixed-effect model was applicable. Statistical significance was defined as a p-value less than 0.05. Funnel plot was employed to assess the presence of publication bias across different research. Ultimately, a sensitivity analysis was conducted to assess the influence of different research on the combined findings and to evaluate the dependability of the results.

## 3 Result

### 3.1 Search results

**Figure 1:**
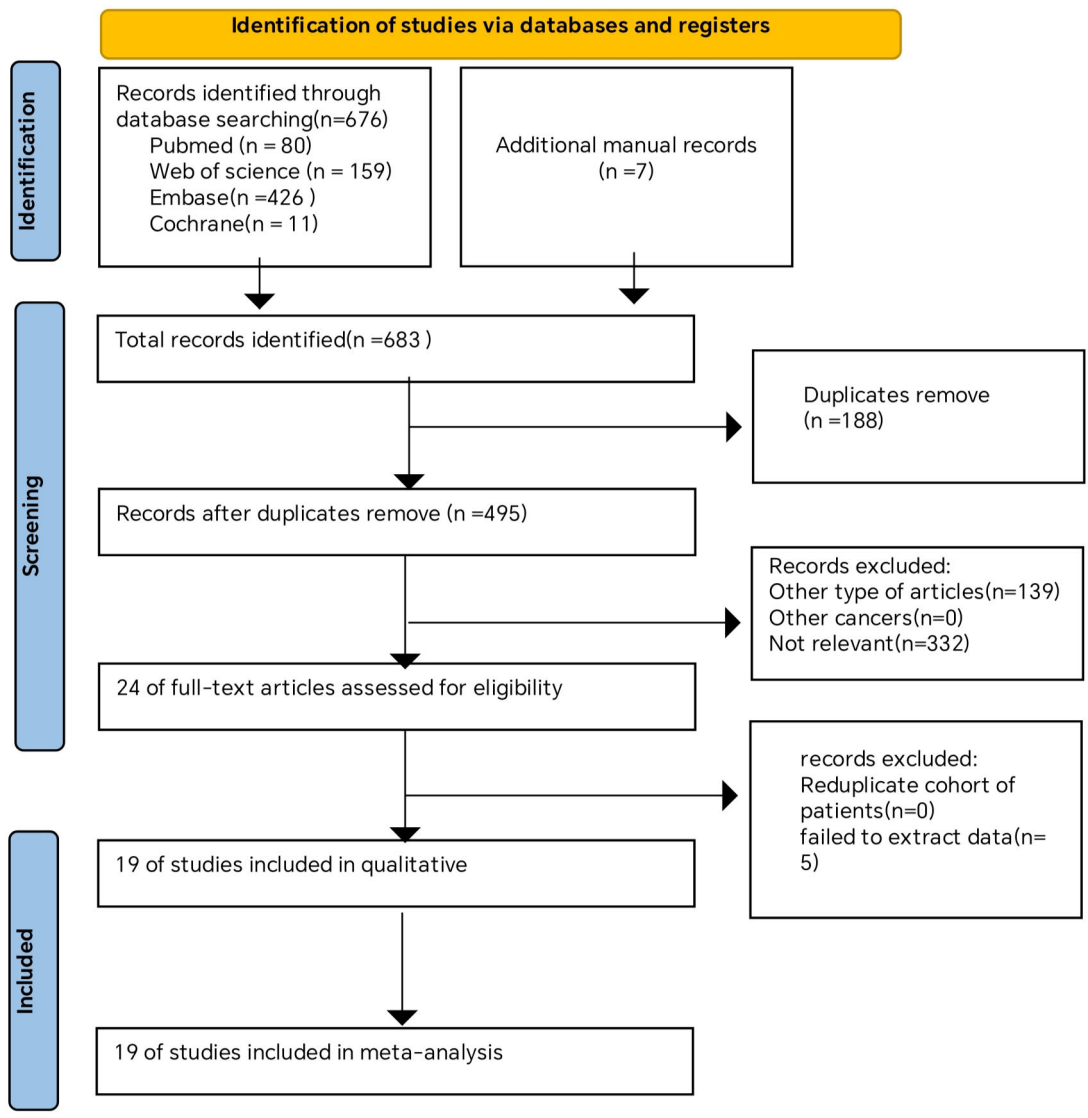
Flow chart of literature search strategies.

A comprehensive overview of the procedure of selecting and integrating literature is provided in the **Figure 1**. Our preliminary search yielded a grand total of 683 papers. Once repeat studies were eliminated, the remaining number of studies was only 495. Following an analysis of the titles and abstracts, a grand total of 471 articles were determined to be irrelevant and so eliminated. After conducting a comprehensive study of the entire text, a final selection of 19 articles (27, 30, 31, 39–46) was made for use in this meta-analysis.

### 3.2 Patient characteristics and quality assessment

A comprehensive summary of the patient characteristics is provided in **Table 1**. This meta-analysis comprised a total of 2002 individuals diagnosed with intermediate or advanced HCC selected from 19 studies published between 2022 and 2025. All studies included were retrospective cohort studies. Within the patient population, 36.6% of the cancers were classified as BCLC stage B, whereas 67.9% were recognized as stage C. An extensive array of PD-1 inhibitors, including as toripalimab, camrelizumab, pembrolizumab, sintilimab, tislelizumab, and nivolumab, were employed in these studies. The intervention of interest in all the studies considered was the concurrent administration of TACE, lenvatinib, and PD-1 inhibitors. In these studies, the control groups received a combination of TACE-L. All these studies were conducted in China. The authors, year, subgroups, regimens, patients, age, ECOG PS (%), Child-Pugh class and BCLC stage of the included literature are shown in **Table 1**. Regarding quality assessment, every study included in the analysis obtained a NOS score superior to 7 points, thereby indicating a high level of quality (**Table 2**).

**Table 1:** Patient characteristics of included studies and patients.

Author, year	Region	Subgroups	Age (mean ± SD, y)	Number of patients	Regimens	ECOG PS (%)		Child-Pugh class		BCLC stage	
						0	1	A	B	B	C
Cai, M.2022 (31)	China	TACE-L-P	51.9 ± 10.3	41	Len: 12 mg (bodyweight ≥60 kg) or 8 mg (bodyweight <60 kg); po; qd. TACE: 20–60 mg Pirarubicin Sin or Tis or Cam: 200 mg; iv; q3w.	33 (80.5)	8 (19.5)	37 (90.2)	4 (9.8)	NA	NA
		TACE-L	54.6 ± 11.0	40	Len: 12 mg (bodyweight ≥60 kg) or 8 mg (bodyweight <60 kg); po; qd. TACE: 20–60 mg Pirarubicin	28 (70.0)	12 (30.0)	33 (82.5)	7 (17.5)	NA	NA
Chen, S.2022 (47)	China	TACE-L-P	≥50: 38 <50: 32	70	Len: 8 mg; po; qd. TACE: Pharmorubicin Pem: 200 mg; iv; q3w.	27 (38.6)	43 (61.4)	NA	NA	47 (67.1)	23 (32.9)
		TACE-L	≥50: 36 (50.0) <50: 36 (50.0)	72	Len: 8 mg; po; qd. TACE: Pharmorubicin	30 (41.7)	42 (58.3)	NA	NA	45 (62.5)	27 (37.5)
Guo, P. 2022 (39)	China	TACE-L-P	>60: 15 (20.0) ≤60: 60 (80.0)	75	Len: 12 mg (bodyweight > 60 kg) or 8 mg (bodyweight < 60 kg); po; qd. TACE: 50 mg/m^2^ Epirubicin Sin or Cam: 200 mg; iv; q3w.	69 (92.0)	6 (8.0)	73 (97.3)	2 (2.7)	20 (26.7)	55 (73.3)
		TACE-L	>60: 25 (27.5) ≤60: 66 (72.5)	91	Len: 12 mg (bodyweight > 60 kg) or 8 mg (bodyweight < 60 kg); po; qd. TACE: 50 mg/m^2^ Epirubicin	58 (63.7)	33 (36.3)	78 (85.7)	13 (14.3)	20 (22.0)	71 (78.0)
Jingzheng, H.2022 (40)	China	TACE-L-P	≥50: 26(59.1) <50: 33(66.0)	44	Len: 12 mg (bodyweight ≥60 kg) or 8 mg (bodyweight <60 kg); po; qd. TACE: 20–60 mg Pirarubicin Sin or Tis or Cam: 200mg; iv; q3w.	35 (79.5)	9 (20.5)	33 (75.0)	11 (25.0)	9 (20.5)	35 (79.5)
		TACE-L	≥50: 18(40.9) <50: 17(34.0)	50	Len: 12 mg (bodyweight ≥60 kg) or 8 mg (bodyweight <60 kg); po; qd. TACE: 20–60 mg Pirarubicin	41 (82.0)	9 (18.0)	38 (76.0)	12 (24.0)	11 (22.0)	39 (78.0)
Qu, W. F.2022 (41)	China	TACE-L-P	55.5 (47.8, 64.3)	30	Len: 8 mg; po; qd. TACE: 50 mg Lobaplatin Tor:240 mg; iv; q3w.	NA	NA	28 (93.3)	2 (6.7)	1 (3.3)	29 (96.7)
		TACE-L	50.0 (45.0, 61.0)	21	Len: 8 mg; po; qd. TACE: 50 mg Lobaplatin	NA	NA	21 (100.0)	0 (0.0)	3 (14.3)	18 (85.7)
Zhao, S.2022 (48)	China	TACE-L-P	52.83 ± 7.14	23	Len: 8 mg (bodyweight< 60 kg) or 12 mg (bodyweight>60 kg); po; qd. TACE: NA Niv or Tor: 3 mg/kg; iv; q2w.	12 (52.2)	11 (47.8)	19 (82.6)	4 (17.4)	6 (26.1)	17 (73.9)
		TACE-L	57.38 ± 9.44	32	Len: 8 mg (bodyweight< 60 kg) or 12 mg (bodyweight>60 kg); po; qd. TACE: NA	18 (56.3)	14 (43.7)	27 (84.4)	5 (15.6)	13 (40.6)	19 (59.4)
Lin, Long Wang 2023 (42)	China	TACE-L-P	57.0 ± 6.4	45	Len: 8 mg (bodyweight< 60 kg) or 12 mg (bodyweight>60 kg); po; qd. TACE: NA Cam: 200 mg; iv; q3w.	NA	NA	28 (62.2)	17 (37.8)	NA	NA
		TACE-L	56.2 ± 11.5	50	Len: 8 mg (bodyweight< 60 kg) or 12 mg (bodyweight>60 kg); po; qd. TACE: NA	NA	NA	25 (50.0)	25 (50.0)	NA	NA
Wang, Wei-Jun 2023 (30)	China	TACE-L-P	57.0 ± 9.9	54	Len: 12 mg (bodyweight ≥60 kg) or 8 mg (bodyweight <60 kg); po; qd. TACE: NA Sin or Cam: 200 mg; iv; q3w; or Tor: 240 mg; iv; q3w.	46 (85.2)	8 (14.8)	49 (90.7)	5 (9.3)	NA	NA
		TACE-L	60.8 ± 9.4	45	Len: 12 mg (bodyweight ≥60 kg) or 8 mg (bodyweight <60 kg); po; qd. TACE	41 (91.1)	4 (8.9)	42 (93.3)	3 (6.7)	NA	NA
Wang, Y. Y.2023 (46)	China	TACE-L-P	≥65: 7 (15.56) <65: 38 (84.44)	45	Len: 8 mg (bodyweight< 60 kg) or 12 mg (bodyweight>60 kg); po; qd. TACE: 25–40 mg/m^2^ pirarubicin Cam or Sin or Pem or Tis: 200 mg; or Tor: 240 mg; iv; q3w; or Niv:240 mg; iv; q2w.	26 (57.8)	19 (42.2)	30 (66.7)	NA	NA	34 (75.6)
		TACE-L	≥65: 8 (40.00) <65: 12 (60.00)	20	Len: 8 mg (bodyweight< 60 kg) or 12 mg (bodyweight>60 kg); po; qd. TACE: 25–40 mg/m^2^ pirarubicin	7 (35.0)	13 (65.0)	18 (90.0)	NA	NA	15 (75.0)
Xiang, Zhanwang 2023 (43)	China	TACE-L-P	>60: 10(30.3) ≤60: 23(69.7)	33	Len: 12 mg (bodyweight ≥60 kg) or 8 mg (bodyweight <60 kg); po; qd. TACE: 20–50 mg Epirubicin hydrochloride Cam: 3 mg/kg; iv; q3w.	22 (66.7)	11 (33.3)	25 (75.8)	8 (24.2)	10 (30.3)	23 (69.7)
		TACE-L	>60: 14(28.6) ≤60: 35(71.4)	49	Len: 12 mg (bodyweight ≥60 kg) or 8 mg (bodyweight <60 kg); po; qd. TACE: 20–50 mg Epirubicin hydrochloride	38 (77.6)	11 (22.4)	41 (83.7)	8 (16.3)	22 (44.9)	27 (55.1)
Yang, H.2023 (27)	China	TACE-L-P	61.4 ± 9.3	64	Len: 8 mg (bodyweight< 60 kg) or 12 mg (bodyweight>60 kg); iv; q3w. TACE: Doxorubicin Sin or Tis or Cam: 200 mg; iv; q3w.	12 (20.7)	46 (79.3)	38 (59.4)	26 (40.6)	26 (40.6)	38 (59.5)
		TACE-L	63.2 ± 8.5	58	Len: 8 mg (bodyweight< 60 kg) or 12 mg (bodyweight>60 kg); po; q3w. TACE: Doxorubicin	11 (17.2)	53 (82.8)	33 (56.9)	25 (43.1%)	24 (41.4%)	34 (58.6%)
Zou, X.2023 (44)	China	TACE-L-P	53.6 ± 15.1	70	Len: 12 mg (bodyweight ≥60 kg) or 8 mg (bodyweight <60 kg); po; qd. TACE: Epirubicin 40 mg Pem or Sin: 200 mg; iv; q3w.	17 (24.3)	53 (75.7)	46 (65.7)	24 (34.3)	NA	70 (100.0)
		TACE-L	52.3 ± 14.8	79	Len: 12 mg (bodyweight ≥60 kg) or 8 mg (bodyweight <60 kg); po; qd. TACE: 40 mg Epirubicin	28 (31.1)	62 (68.9)	61 (67.8)	29 (32.2)	NA	90 (100.0)
Sheng, Y. 2024 (45)	China	TACE-L-P	64.48 ± 10.83	113	Len: 12mg (≥60kg bodyweight) or 8mg (<60 kg bodyweight); po; qd. TACE: Pirarubicin Sin or Tis or Cam: 200mg; po; q3w.	62 (54.9)	36 (31.9)	88 (77.9)	25 (22.1)	54 (47.8)	59 (52.2)
		TACE-L	62.59 ± 10.58	128	Len: 12mg (≥60kg bodyweight) or 8mg (<60 kg bodyweight); po; qd. TACE: Pirarubicin	66 (51.6)	42 (32.8)	99 (77.3)	29 (22.7)	63 (49.2)	65 (50.8)
Wu, H. X.2024 (49)	China	TACE-L-P	56.9 ± 8.1	18	Len: 12mg (≥60kg bodyweight) or 8mg (<60 kg bodyweight); po; qd. TACE: 20mg/m^2^ Lobaplatin Sin or Cam or Niv or Tis: 200mg; iv; q3w.	7 (38.9)	11 (61.1)	18 (100.0)	0 (0.0)	NA	NA
		TACE-L	58.1 ± 9.4	23	Len: 12mg (≥60kg bodyweight) or 8mg (<60 kg bodyweight); po; qd. TACE Lobaplatin 20mg/m^2^	7 (30.4)	16 (69.6)	21 (91.3)	2 (8.7)	NA	NA
Chen, Song.2024 (50)	China	TACE-L-P	55.8 ± 11.2	66	Len: 12mg (≥60kg bodyweight) or 8mg (<60 kg bodyweight);po;qd. TACE: microspheres;gelatin sponge particles Tis:iv;q3w.	NA	NA	59 (89.4)	7 (10.6)	NA	NA
		TACE-L	56.6 ± 12.1	45	Len: 12mg (≥60kg bodyweight) or 8mg (<60 kg bodyweight);po;qd. TACE: microspheres; gelatin sponge particles	NA	NA	40 (88.9)	5 (11.1)	NA	NA
Ding, Zongren. 2024 (51)	China	TACE-L-P	57.0 [47.0, 64.5]	19	Len: 12mg (≥60kg bodyweight) or 8mg (<60 kg bodyweight);po;qd. TACE: epirubicin; iodine oil Cam or Tis or Sin or Pem: iv;q3w.	3	16	13	6	5	13
		TACE-L	61.5 [53.0, 65.0]	16	Len: 12mg (≥60kg bodyweight) or 8mg (<60 kg bodyweight);po;qd. TACE: epirubicin;iodine oil	0	16	10	6	5	10
Jiang, J.2024 (52)	China	TACE-L-P	55.3 ± 9.1	68	Len: 12mg (≥60kg bodyweight) or 8mg (<60 kg bodyweight);po.qd. TACE: Lipiodol: 2–20 mL;epirubicin: 20–60 mg;polyvinyl alcohol particles Tis: 200 mg; iv; q3w.	54 (79.4)	14 (20.6)	61 (89.7)	7 (10.3)	23 (33.8)	45 (66.2)
		TACE-L	55.2 ± 12.3	68	Len: 12mg (≥60kg bodyweight) or 8mg (<60 kg bodyweight);po.qd. TACE: Lipiodol: 2–20 mL;epirubicin: 20–60 mg;polyvinyl alcohol particles	53 (77.9)	15 (22.1)	59 (86.8)	9 (13.2)	22 (32.4)	46 (67.6)
Zhao, Y.2024 (53)	China	TACE-L-P	≤70:90 >70:13	103	Len: 12mg (≥60kg bodyweight) or 8mg (<60 kg bodyweight);po.qd. TACE: Iodized oil;epirubicin (50 mg/m2);gelatin sponge particles Tis: 200 mg; iv; q3w.	NA	NA	90 (82.57%)	19 (17.43%)	17 (16.5%)	86 (83.5%)
		TACE-L	≤70:54 >70:12	66	Len: 12mg (≥60kg bodyweight) or 8mg (<60 kg bodyweight);po.qd. TACE: Iodized oil;epirubicin (50 mg/m2);gelatin sponge particles	NA	NA	57 (83.82%)	11 (16.18%)	11 (16.7%)	55 (83.3%)
Wu, F. D.2025 (54)	China	TACE-L-P	≥60: 15 < 60:15	30	Len: 12mg (≥60kg bodyweight) or 8mg (<60 kg bodyweight);po.qd. TACE: epirubicin;iodised oil;gelatin sponge particles Sin: 200 mg; iv; q3w.	8	22	27	3	15	15
		TACE-L	≥60: 16 < 60:11	27	Len: 12mg (≥60kg bodyweight) or 8mg (<60 kg bodyweight);po.qd. TACE: epirubicin;iodised oil;gelatin sponge particles	7	20	25	2	22	5

SD, standard deviation; BCLC, Barcelona Clinic Liver Cancer; ECOG, Eastern Cooperative Oncology Group; Len, lenvatinib;TACE, transarterial chemoembolization; PD-1, programmed death-1; Len, Lenvatinib; Sin, Sintilimab; Tis, Tislelizumab, Cam, Camrelizumab; Pem, Pembrolizumab; Tor, Toripalimab; Niv, Nivolumab; NR, not reported; qd, once a day; q3w, every 3 weeks; q2w, every 2 weeks; po, administered orally; iv, intravenous injection.

**Table 2:** Quality assessment according to the NOS scale.

Author, year	Selection				Comparability		Outcome			Total scores
	Representativeness	Selection of non-exposure	Ascertainment of exposure	Outcome not present at start	Comparability on most important factors	Comparability on other risk factors	Assessment of outcome	Adequate follow-up time	Complete follow-up	
Cai, M.2022 (31)	*	*	*	*	*	*	*	*	*	9
Chen, S.2022 (47)	*	*	*	*	*	–	*	*	*	8
Guo, P. 2022 (39)	*	*	*	*	*	–	*	*	*	8
Jingzheng, H.2022 (40)	*	*	*	*	*	–	*	–	*	7
Qu, W. F.2022 (41)	*	*	*	*	*	*	*	*	*	9
Zhao, S.2022 (48)	*	*	*	*	*	–	*	–	*	7
Lin, Long Wang 2023 (42)	*	*	*	*	*	*	*	*	*	9
Wang, Wei-Jun 2023 (30)	*	*	*	*	*	–	*	–	*	7
Wang, Y. Y.2023 (46)	*	*	*	*	*	–	*	*	*	8
Xiang, Zhanwang 2023 (43)	*	*	*	*	*	–	*	–	*	7
Yang, H.2023 (27)	*	*	*	*	*	–	*	*	*	8
Zou, X.2023 (44)	*	*	*	*	*	–	*	*	*	8
Sheng, Y. 2024 (45)	*	*	*	*	*	*	*	*	*	9
Wu, H. X.2024 (49)	*	*	*	*	*	*	*	*	*	9
Chen, Song.2024 (50)	*	*	*	*	*	–	*	–	*	7
Ding, Zongren.2024 (51)	*	*	*	*	*	–	*	*	*	8
Jiang, J.2024 (52)	*	*	*	*	*	*	*	–	–	7
Zhao, Y.2024 (53)	*	*	*	*	*	*	*	*	*	9
Wu, F. D.2025 (54)	*	*	*	*	*	*	*	*	*	9

NOS, Newcastle-Ottawa scale; “*”indicates criterion met; “-”indicates significant of criterion not.

### 3.3 Efficacy outcomes

#### 3.3.1 ORR and DCR

**Figure 2:**
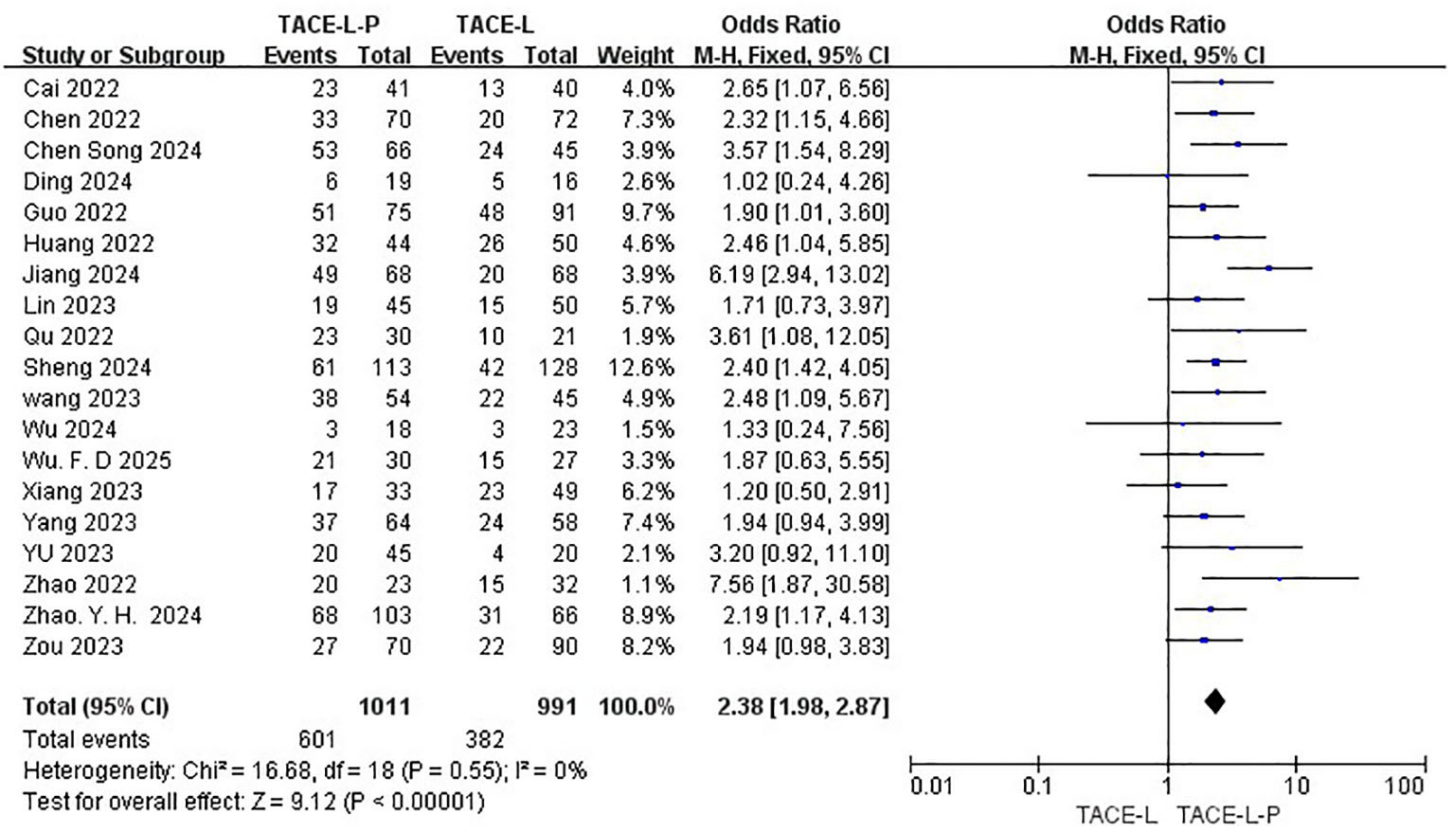
Forest plot of the meta-analysis for ORR.

**Figure 3:**
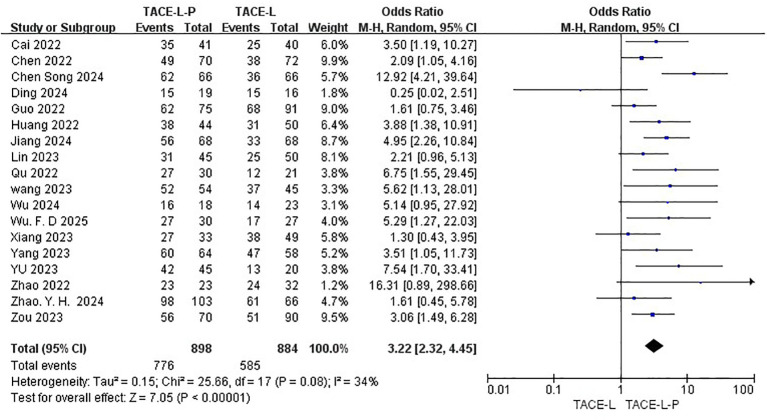
Forest plot of the meta-analysis for DCR.

All 19 studies included reported data on ORR (27, 30, 31, 40–48), while only 18 studies provided DCR (27, 30, 31, 39–43, 46–49). The ORR of patients in the TACE-L-P group was significantly greater than that of the TACE-L group (59.45% vs 38.55%, OR = 2.38, 95% CI: 1.98 to 2.87, P <0.00001, I^2^ = 0%) (**Figure 2**). Sensitive analysis showed that the outcomes were stable (**Supplementary Figure S1**). The DCR of patients in the TACE-L-P group was also significantly better than that of the TACE-L group (86.41% vs 66.18%, OR = 3.22, 95% CI: 2.32 to 4.45, p <0.00001, I^2^ = 0%) (**Figure 3**). Sensitive analysis showed that the outcomes were stable (**Supplementary Figure S2**).

#### 3.3.2 PFS and OS

**Figure 4:**
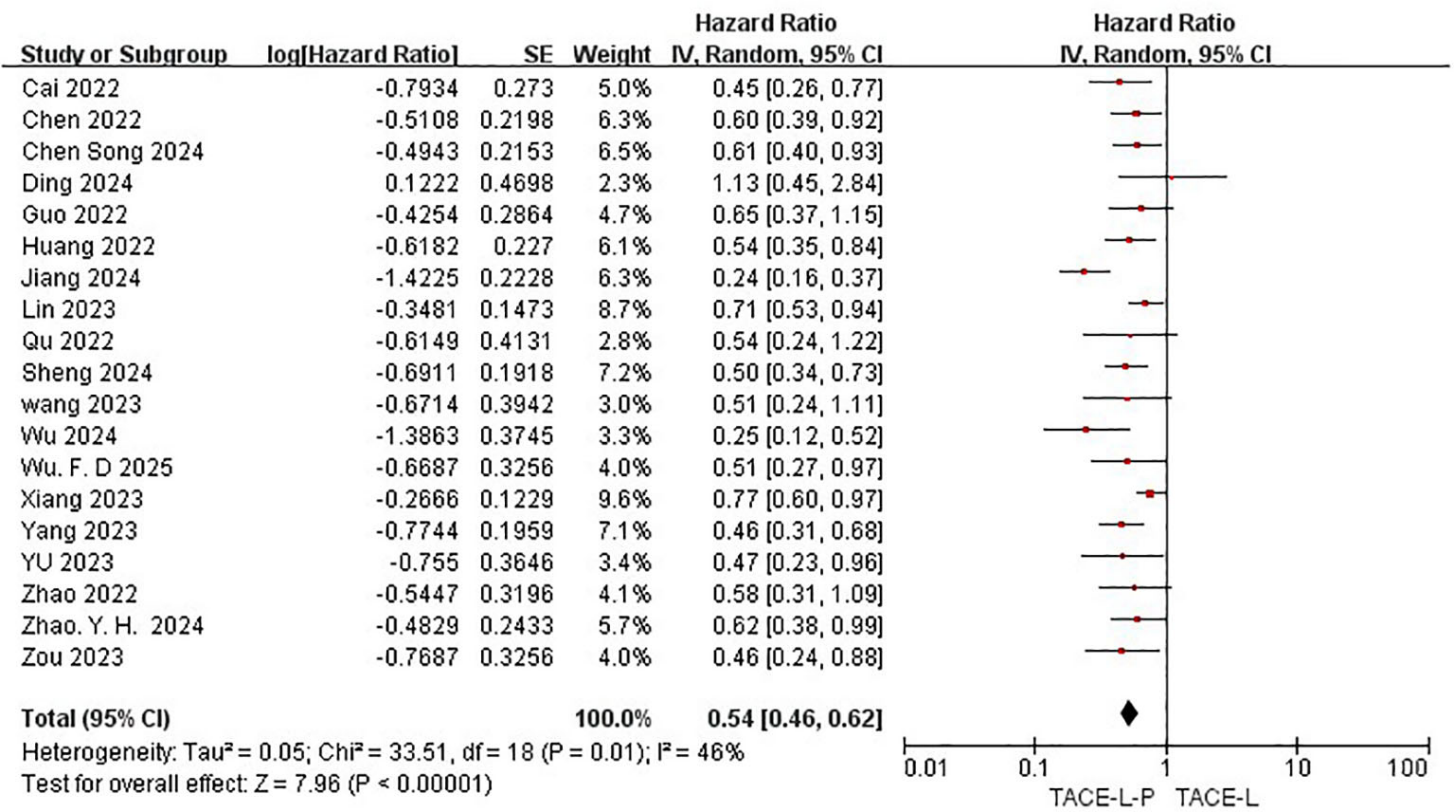
Forest plot of the meta-analysis for PFS.

**Figure 5:**
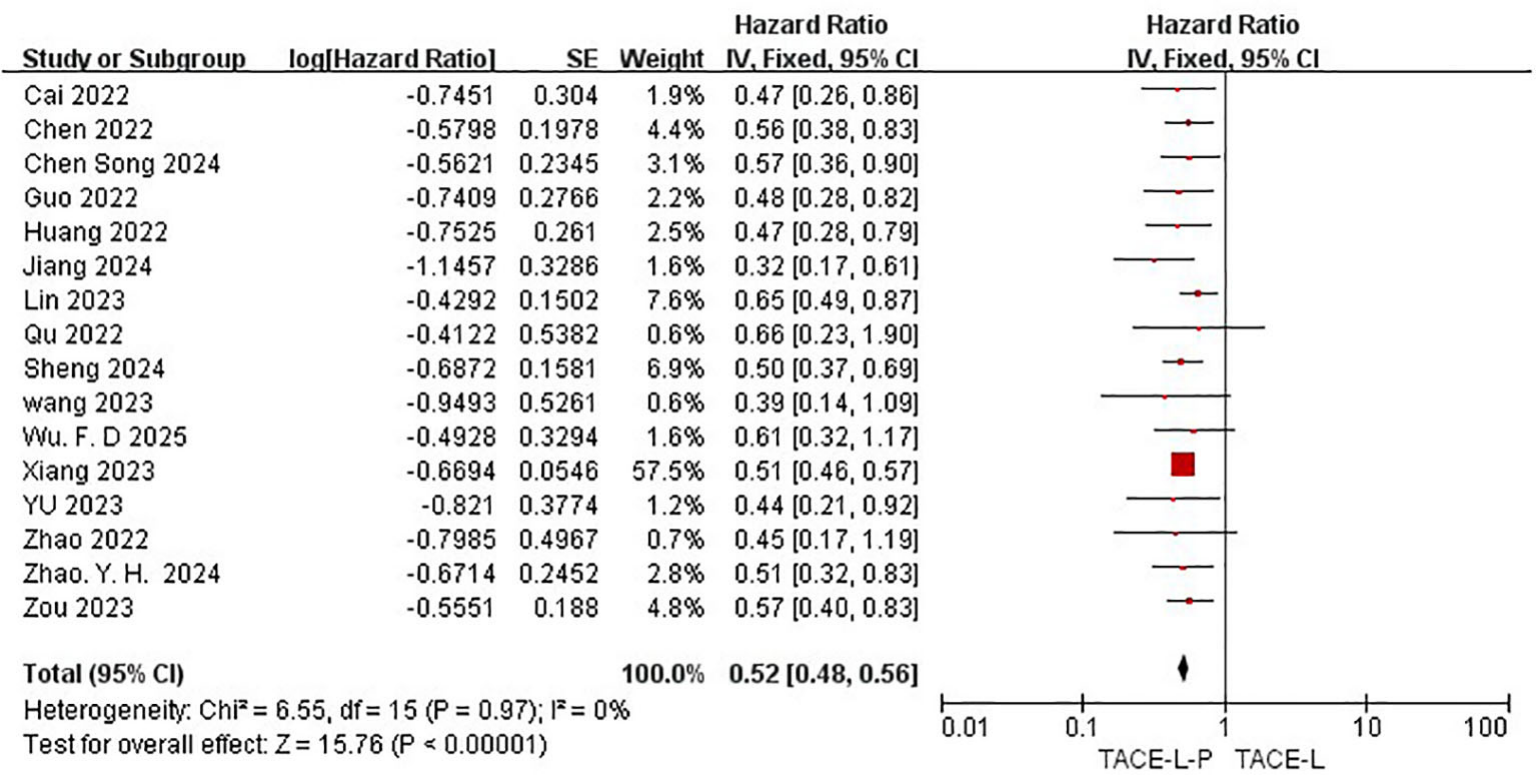
Forest plot of the meta-analysis for OS.

All 19 studies included in the meta-analysis provided KM curves for PFS (27, 30, 31, 39–54), while only 16 studies provided KM curves for OS (30, 31, 39–42, 45–47, 50, 52). TACE-L-P emerged as superior to TACE-L in terms of PFS (HR: 0.54, 95%CI 0.46 to 0.62, P<0.00001, I^2^ = 46%) (**Figure 4**) and OS (HR: 0.52, 95%CI 0.48 to 0.56, P<0.0001, I^2^ = 0%) (**Figure 5**). Sensitive analysis showed that the outcomes were stable (**Supplementary Figures S3**, **S4**).

**Figure 6:**
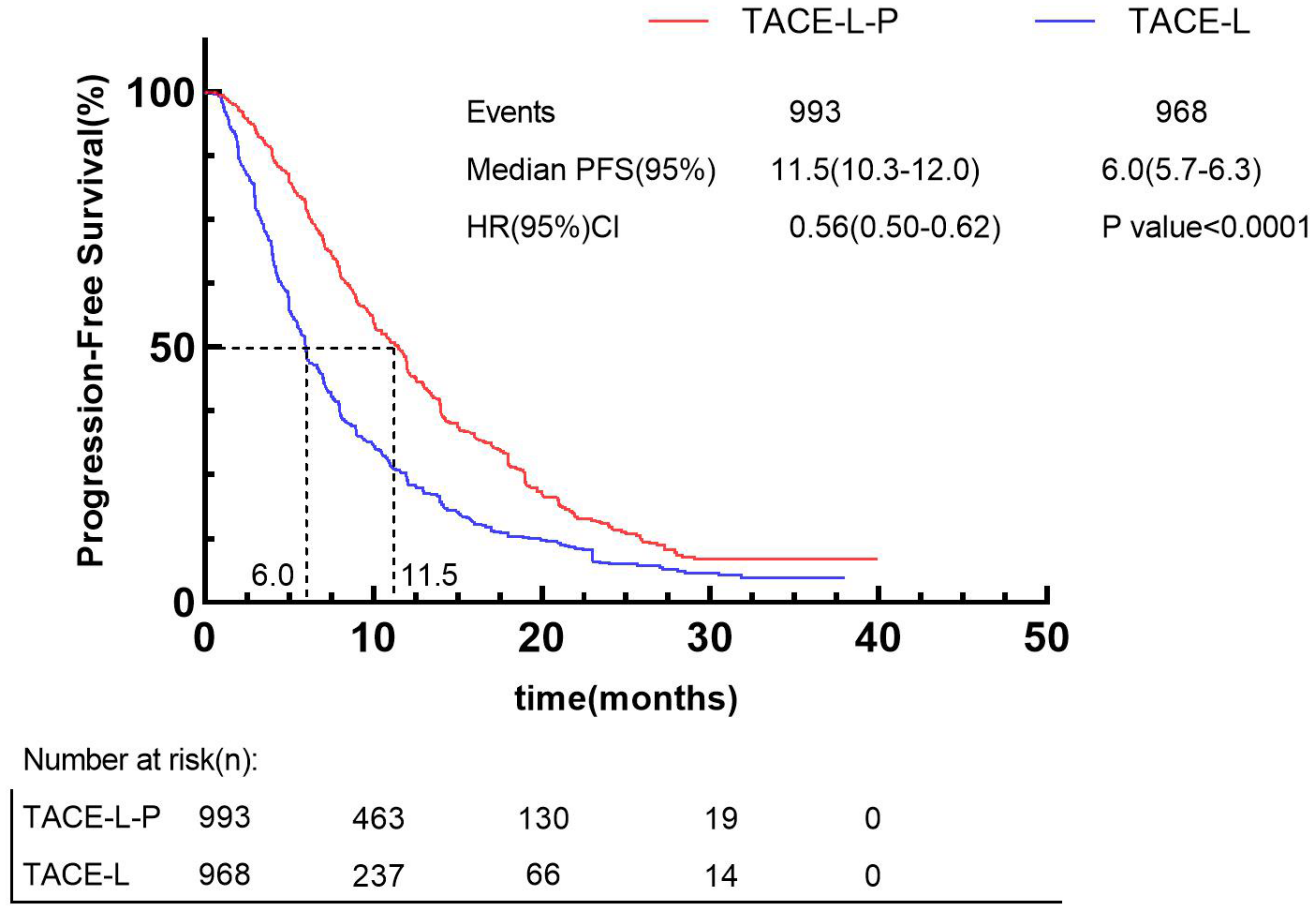
Kaplan-Meier curves for PFS.

**Figure 7:**
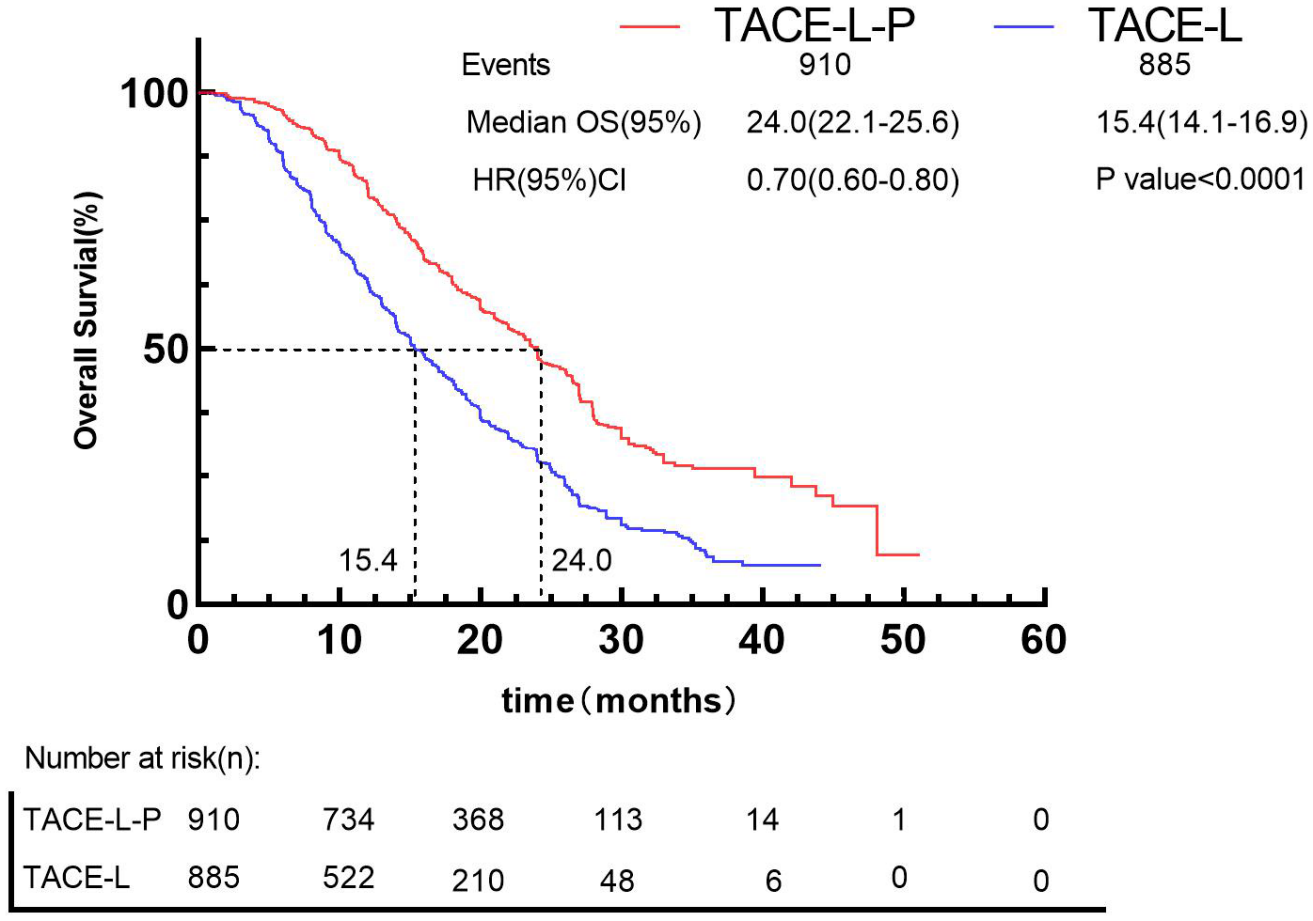
Kaplan-Meier curves for OS.

Besides, by using the IPDformKM program, reconstruction of Kaplan-Meier curves provided a clear and comprehensible representation of oncological outcomes for PFS (median survival time: 11.5 months versus 6.0 months, HR: 0.56, 95%CI 0.50 to 0.62, P<0.0001) (**Figure 6**) and OS (median survival time: 24.0 months versus 15.4 months, HR: 0.70, 95%CI 0.60 to 0.80, P<0.0001) (**Figure 7**).

### 3.4 Safety

**Figure 8:**
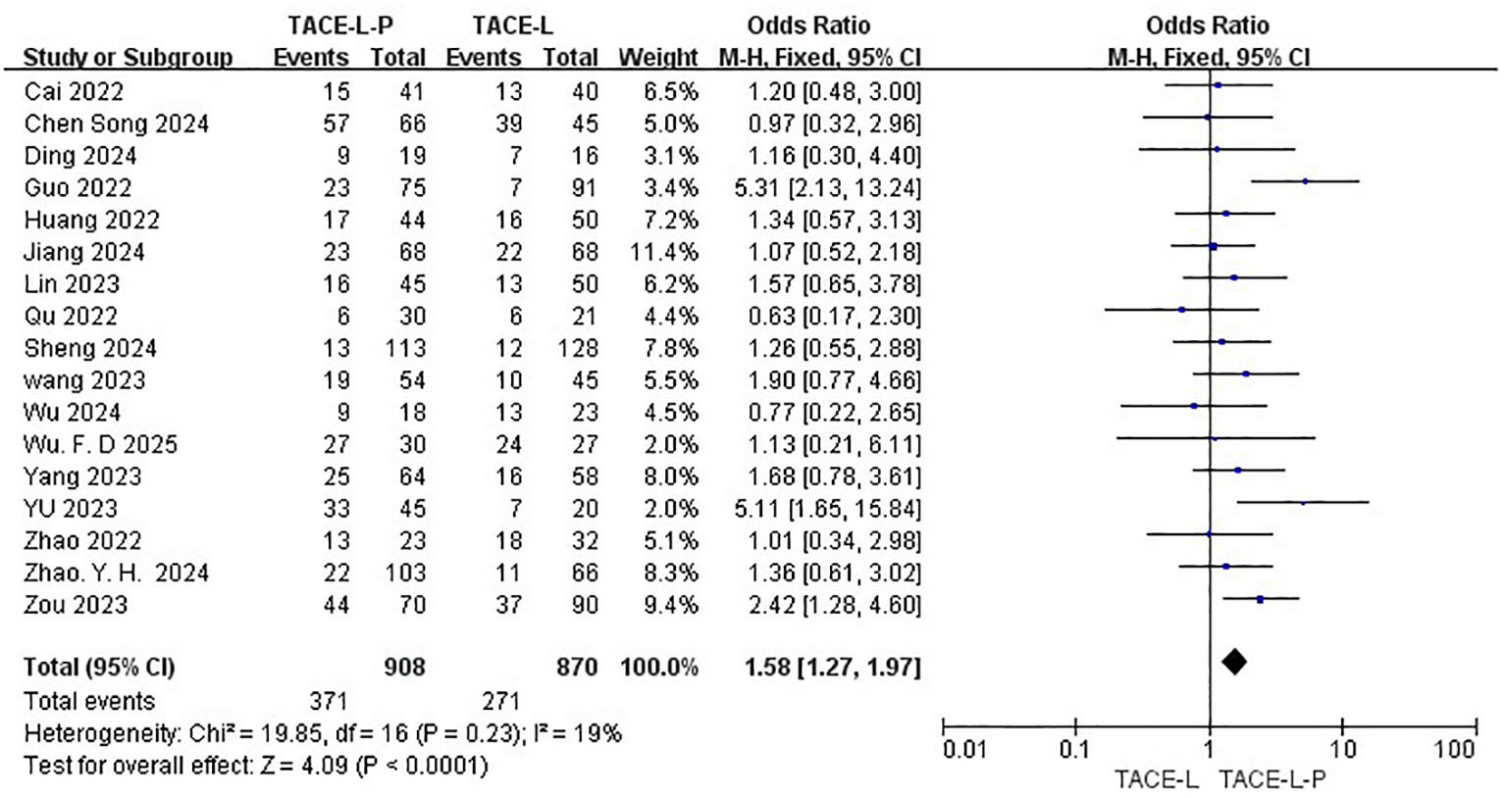
Forest plot of the meta-analysis for Grade≥ 3 AEs rate.

Safety was assessed by evaluating the rate of Grade≥ 3 AEs reported in a total of 17 studies (27, 30, 31, 39–46, 48–54). The TACE-L-P group showed a higher probability of experiencing Grade≥ 3 AEs compared to the TACE-L group(40.86% vs 31.15%, OR=1.58, 95% CI: 1.27 to 1.97, P<0.0001, I^2^ = 19%) (**Figure 8**). Sensitive analysis showed that the outcomes were stable (**Supplementary Figure S5**).

### 3.5 Publication bias

Publication bias was assessed by analyzing a funnel plot in connection to the ORR (**Supplementary Figure S6**), DCR (**Supplementary Figure S7**), PFS (**Supplementary Figure S8**), OS (**Supplementary Figure S9**) and Grade≥ 3 AEs (**Supplementary Figure S10**). The bilateral symmetric funnel plots indicated that there was no substantial evidence of publication bias.

### 3.6 Subgroup analyses for individual Grade≥3 AEs

Subgroup analyses for individual Grade≥3 AEs were performed (**Table 3**, **Supplementary Figures S11–S22**). TACE-L-P significantly increased the incidence of hypertension (OR 1.72, 95% CI 1.03–2.20; P = 0.03) and rash (OR 2.00, 95% CI 0.88–4.53; P = 0.02) compared with TACE-L. There was no statistically significant differences between two groups regarding decreased appetite, elevated AST, elevated ALT, fatigue, diarrhea, abdominal pain, hand-foot syndrome, thrombocytopenia, hypothyroidism or fever.

**Table 3:** Subgroup analysis for individual Grade≥3 AEs.

Grade≥ 3 AEs	No of studies	TACE-L-P	TACE-L	RR[95%CI]	P value
Decreased appetite	9	4.40%	3.36%	1.32 [0.71, 2.45]	0.38
Elevated AST	12	10.80%	9.92%	1.01 [0.69, 1.48]	0.95
Elevated ALT	12	9.96%	7.80%	1.20 [0.81, 1.77]	0.36
Fatigue	12	5.10%	3.17%	1.72 [0.99, 2.98]	0.06
Diarrhoea	12	5.42%	3.70%	1.35 [0.81, 2.24]	0.25
Abdominal pain	10	4.48%	4.10%	1.09 [0.60, 1.97]	0.77
Hypertension	16	9.23%	6.52%	1.51 [1.03, 2.20]	0.03
Rash	9	5.54%	2.51%	2.14 [1.10, 4.14]	0.03
Hand-foot syndrome	12	5.06%	3.54%	1.45 [0.87, 2.41]	0.15
Thrombocytopenia	9	4.80%	4.29%	1.11 [0.59, 2.11]	0.74
Hypothyroidism	7	4.42%	1.91%	2.00 [0.88, 4.53]	0.09
Fever	5	2.91%	3.41%	0.82 [0.34, 1.99]	0.67

TACE, transarterial chemoembolization; L, lenvatinib; P, programmed cell death 1 inhibitor; AST, aspartate aminotransferase; ALT, alanine aminotransferase; No., number; RR, relative risk; CI, confidence intervals.

### 3.7 Subgroup analysis regarding ORR, DCR, PFS and OS for individual PD-1 Inhibitors

Different PD-1 Inhibitors were employed among the included 19 studies. camrelizumab was employed in two studies, tislelizumab was employed in three studies, pembrolizumab was employed in one study, toripalimab was employed in one study and sintilimab was employed in one study. However, more than one type of PD-1 Inhibitors were employed in another 11 studies and the outcomes data were undistinguishable for individual PD-1 Inhibitors (**Table 1**, regimens). Subgroup analyses regarding ORR, DCR, PFS and OS for individual PD-1 Inhibitors were performed (**Table 4**, **Supplementary Figures S23–S26**). The ORR of patients in the TACE-L-P group was significantly greater than that of the TACE-L group when tislelizumab, pembrolizumab or toripalimab was employed. The DCR of patients in the TACE-L-P group was significantly greater than that of the TACE-L group when tislelizumab, pembrolizumab, sintilimab or toripalimab was employed. The PFS of patients in the TACE-L-P group was significantly better than that of the TACE-L group when tislelizumab, pembrolizumab or camrelizumab was employed. The OS of patients in the TACE-L-P group was significantly better than that of the TACE-L group when tislelizumab, pembrolizumab or camrelizumab was employed.

**Table 4:** Results of Subgroup analysis for PD-1.

PD-1 Inhibitor	ORR				DCR				PFS				OS			
	No.	OR (95%CI)	P	I^2^ (%)	No.	OR (95%CI)	P	I^2^ (%)	No.	HR (95%CI)	P	I^2^ (%)	No.	HR (95%CI)	P	I^2^ (%)
Camrelizumab	2	1.44 (0.78-2.66)	0.24	0%	2	1.83 (0.93-3.57)	0.08	0%	2	0.74 (0.62-0.89)	0.001	0%	2	0.55 (0.44-0.69)	<0.00001	56%
Tislelizumab	3	3.56 (1.90-6.66)	<0.0001	54%	3	4.87 (1.73-13.68)	0.003	66%	3	0.59 (0.40-0.87)	0.007	70%	3	0.48 (0.35-0.66)	<0.00001	8%
Pembrolizumab	1	2.32 (1.15-4.66)	0.02	/	1	2.09 (1.05-4.16)	0.04	/	1	0.56 (0.38-0.83)	0.003	/	1	0.60 (0.39-0.92)	0.02	/
Toripalimab	1	3.61 (1.08-12.05)	0.04	/	1	6.75 (1.55-29.45)	0.01	/	1	0.66 (0.23-1.90)	0.44	/	1	0.54 (0.24-1.22)	0.14	/
Sintilimab	1	1.87 (0.63-5.55)	0.26	/	1	5.29. (1.27-22.03)	0.02	/	1	0.61 (0.32-1.17)	0.13	/	1	0.51 (0.27-0.97)	0.04	/
Undistinguishable PD-1 types	11	2.27 (1.77-2.90)	<0.00001	0%	10	3.03 (2.14-4.30)	<0.00001	19%	8	0.52 (0.42-0.60)	<0.00001	0%	11	0.50 (0.43-0.59)	<0.00001	0%

CI, confidence interval; ORR, Objective Response Rate; DCR, Disease Control Rate; PFS, Progression-free survival; OS, Overall survival; OR, Odds Ratio; HR, Hazard Ratio; PD-1, PD-1 inhibitor.

### 3.8 Evidence certainty

**Figure 9:**
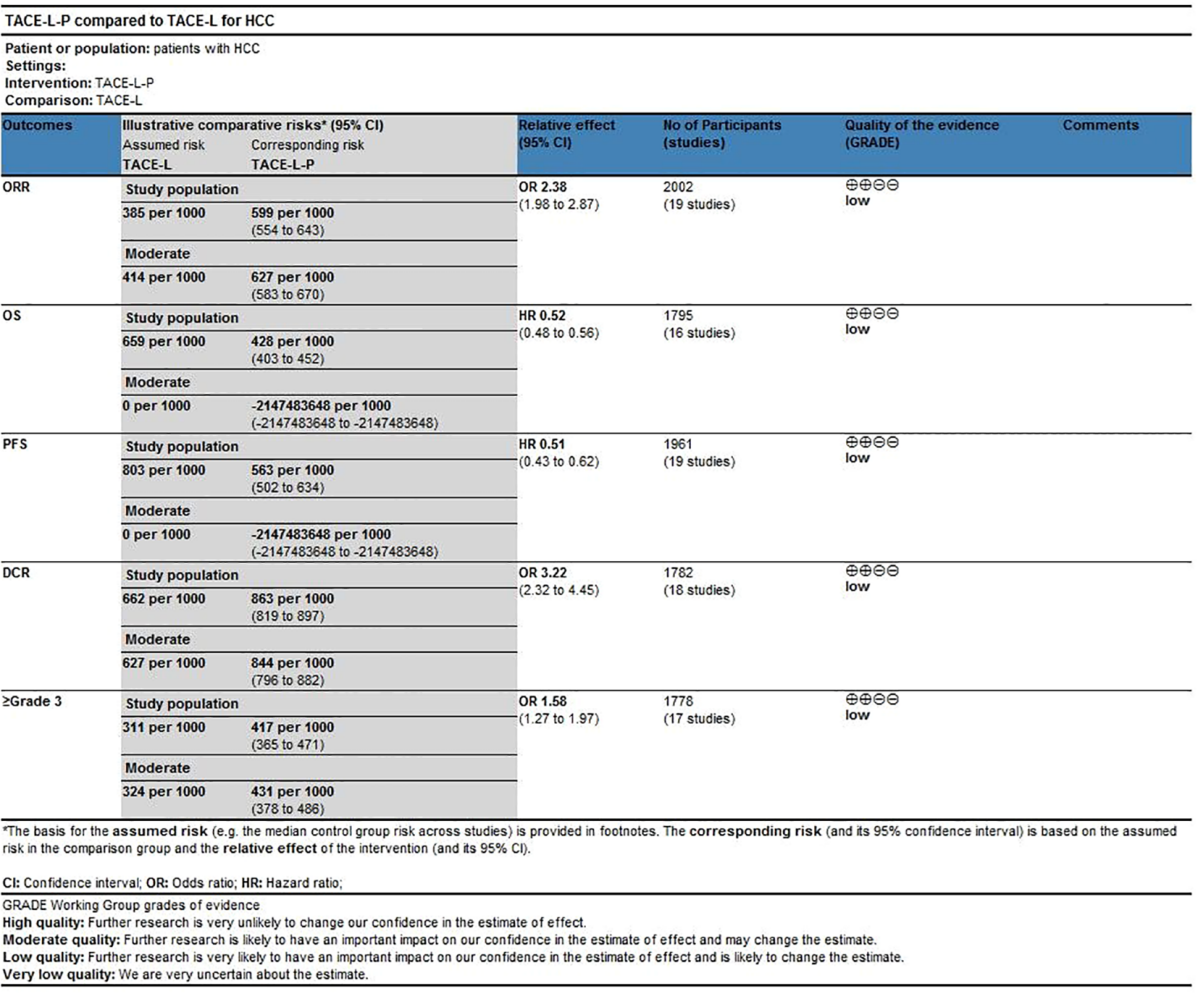
Certainty of evidence using GRADEpro GDT.

The certainty of evidence assessed for the various outcomes as per GRADE (Grading of Recommendations, Assessment, Development and Evaluations) criteria were of low certainty category (**Figure 9**).

## 4 Discussion

Efficacy and safety of TACE-L-P versus TACE-L for intermediate or advanced HCC were compared in this meta-analysis. Our findings indicate that TACE-L-P greatly enhanced ORR, DCR, PFS and OS, with a rise in the probability of Grade≥ 3 AEs. The co-administration of TACE-L has demonstrated a positive outlook for the management of primary liver cancer.

A previous meta-analysis demonstrated that TACE-L increased ORR, DCR, and 6-month, 12-month, and 18-month PFS in patients with advanced HCC, while lowering blood AFP and VEGF expression levels. Compared with TACE alone, TACE-L did not significantly improve 6-month OS, but it significantly improved 12-month and 18-month OS (55). Nevertheless, the co-administration of TACE-L may not be advantageous for many patients, particularly those with extrahepatic metastases, as this may be linked to the process of T cell escape recognition. The ability of cancer cells to evade the immune system may explain why HCC can elude treatment with TACE-L and other conventional therapeutic approaches (56–58). Over the last ten years, significant advancements have been achieved in the systematic therapy of advanced HCC using specific anticancer drugs and ICIs (41). The phase 1b trial, Study 116–KEYNOTE-524, demonstrated that the combination of lenvatinib and pembrolizumab showed impressive antitumor activity in first-line treatment. These patients had a median overall survival of 22.0 months, a median progression-free survival of 8.6 months, and a manageable safety profile (59). Atezolizumab combined with bevacizumab was granted approval by the US Food and Drug Administration in May 2020 as the primary treatment for advanced HCC (60, 61). All these studies suggested the cooperation between local treatment, immunotherapy, and targeted therapy (41). Furthermore, the effectiveness and safety of immune checkpoint inhibitors, such as pembrolizumab and camrelizumab, which block immune evasion through the PD-1/PD-L1 pathway, have been proven in several prior studies for advanced HCC. TACE enhances adaptive immunity by liberating tumor antigens under hypoxia, creating a “vascular-immune priming” condition (62). TACE-L-P combined with PD-1 inhibitors enhances outcomes via synergistic biological interactions. To reinstate T-cell depletion, PD-1 inhibition interferes with the PD-1/PD-L1 axis, whereas lenvatinib rectifies tumor vasculature to enhance immune cell infiltration into the tumor microenvironment (42, 62). These inhibitors appear to counteract the effects of tumor evasion induced by conventional therapy, so serving a supplementary function (63). Mechanistically, the combination therapy creates a “vascular- immune priming” microenvironment: lenvatinib normalizes tumor vasculature to enhance immune cell infiltration, whereas PD-1 inhibitors augment T-cell activity (63–66). This synergy results in improved survival rates: a phase II trial reported a median progression-free survival (PFS) of 8.0 months and overall survival (OS) of 18.4 months in triple therapy groups, far surpassing existing dual therapy statistics (PFS: 5.1 months; OS: 10.7 months) (62). Possible explanations could be as follows: (1) TACE causes significant local tissue death and can thereafter trigger anticancer immune responses that can be enhanced with immunomodulatory PD-1 drugs (67, 68); (2) A multikinase inhibitor with antiproliferative and antiangiogenic properties (69), lenvatinib may prevent hypoxia-induced angiogenesis following TACE (67, 70) and modulate the tumor immune microenvironment to increase immune response to PD-1 inhibitor in HCC (69, 71); (3)Blockade of PD-1 inhibitor impedes the transmission of immune assault signals to tumors, therefore enhancing the immune response against tumour cells (72). TACE can efficiently decrease the blood flow to urine-derived hepatocellular carcinoma (uHCC) and stimulate the secretion of tumor-specific antigens, so improving the therapeutic effectiveness of PD-1 inhibitor (73, 74). Hence, the concurrent use of TACE, lenvatinib, and PD-1 inhibitor may result in a synergistic anticancer effect, hence enhancing clinical outcomes in patients with advanced HCC (31).

The therapeutic advantages of combination treatments may differ among subpopulations. Patients with BCLC-C stage HCC and extrahepatic metastases provide a unique clinical challenge. A recent multicenter investigation revealed that patients with distant metastases have markedly poorer response rates to TACE-L-P compared to those without metastases (ORR 28% vs 52%, p=0.009), likely attributable to systemic immunosuppressive characteristics and tumor heterogeneity in metastatic lesions (75). The diminished efficacy in this category corresponds with the suggested causes of T cell exhaustion and PD-L1 overexpression in circulating tumor cells from metastatic locations (76, 77). Yan et al. reported 52.4% of patients with BCLC-B stage HCC were converted to resectable HCC after the treatment of TACE-L-P. However, none of the included studies provided individual data of BCLC-B1 stage and BCLC-B2 stage. More detailed exploration about BCLC subclassification should be considered in future studies.

The choice of TACE methods and chemotherapeutic drugs may significantly impact the synergistic results with lenvatinib and PD-1 inhibitors. Recent evidence suggests that drug-eluting bead transarterial chemoembolization (DEB-TACE) utilizing doxorubicin-loaded microspheres exhibits enhanced local tumor control relative to conventional lipiodol-based transarterial chemoembolization (cTACE), especially when integrated with systemic therapy (78). A multicenter study comparing cTACE (doxorubicin 50 mg + lipiodol) with DEB-TACE (100-300 μm doxorubicin-loaded microspheres) in conjunction with lenvatinib revealed that DEB-TACE attained superior objective response rates (63% vs. 48%, p=0.02) and extended median progression-free survival (11.2 vs. 8.4 months, p=0.03), presumably attributable to sustained drug release and diminished systemic exposure (79). Moreover, embolic agents may variably influence the immune microenvironment; experimental models demonstrate that 70-150 μm microspheres induce greater tumor necrosis while maintaining peri-tumoral dendritic cells, hence enhancing antigen presentation and subsequent efficacy of PD-1 inhibitors (80). The data indicate that DEB-TACE utilizing calibrated particle size and a doxorubicin dosage of 150 mg (recommended for HCC >5 cm) may serve as an effective foundation for combination therapies (81).

Regarding safety, our findings indicated that the incidence of Grade≥ 3 AEs was 40.86% following TACE-L-P, whereas the incidence of Grade≥ 3 AEs was 31.15% following TACE-L. Considering the improved efficacy of TACE-L-P, median PFS from 6.0 months to 11.5 months (**Figure 7**) and median OS from 15.4 months to 24.0 months (**Figure 8**), the higher incidence of Grade ≥ 3 AEs seems to be acceptable. The occurrence of adverse events is unavoidably heightened by triple or dual therapy, with the most prevalent adverse events being impaired liver function, hypertension, and reduced appetite. The increased occurrence of hypertension in patients may be attributed to the combined impact on angiogenesis. Additionally, the lower appetite is largely caused by the increased toxicities related to the combination therapy (82, 83). The elevated incidence of AEs noted in the TACE-L-P triple therapy group may be ascribed to the overlapping and synergistic toxicities of its constituents. Initially, PD-1 inhibitors can induce immune-related adverse events including hypothyroidism, hepatitis, and pneumonitis as a result of systemic immune activation that affects both neoplastic and normal tissues (84). Second, Lenvatinib, a multi-targeted tyrosine kinase inhibitor (TKI), obstructs VEGF receptors (VEGFR1-3), FGFR, and PDGFRα, leading to hypertension (35% compared to 18% in dual therapy), proteinuria, and hepatic dysfunction via vascular destabilization and metabolic dysregulation (62). Third, TACE-induced hypoxia may aggravate lenvatinib-associated hepatotoxicity by hindering drug clearance in cirrhotic livers, while simultaneously facilitating the release of pro-inflammatory cytokines that enhance immune-mediated toxicity (42). The synergy enhances anticancer activity but also elevates off-target consequences. A phase II trial indicated that grade ≥3 hypertension occurred in 35% of patients treated with TACE-L-P, possibly attributable to endothelial dysfunction generated by VEGF suppression, exacerbated by the vascular toxicity of ICIs (62). The simultaneous administration of TACE and lenvatinib may hinder liver regeneration in cirrhotic patients, resulting in increased transaminases and bilirubin levels (42). Active management techniques, including biomarker-guided patient selection (e.g., excluding Child-Pugh B/C cirrhosis) and sequential administration (commencing TACE prior to PD-1 inhibitors), may alleviate these hazards without diminishing efficacy (84). Future research should investigate pharmacodynamic biomarkers, such as circulating exosomal circCCAR1, to anticipate adverse event susceptibility and refine dosing regimens (85). It is advisable to formulate clinical response strategies for these high-frequency or severe AEs to offer more thorough guidance for clinical applications. Fortunately, most of these patients had relief by lenvatinib dose decrease, cessation of anti-programmed death-1 antibodies, and symptomatic treatment. Hence, both the triple and double combination therapies did not elevate the likelihood of uncontrollable adverse events. The safety outcomes underscore the necessity of educating both healthcare professionals and patients; prompt intervention for adverse events is crucial for effectively treating patients with the combination. Physicians should prioritize monitoring of medication toxicity and liver function following the introduction of the treatment. It is important to analyze the long-term safety profile in more detail.

The choice between triple therapy (TACE-L-P) and dual therapy (TACE-L) for intermediate or advanced HCC should be informed by a thorough evaluation of tumor biology, baseline liver function, and immune-microenvironment attributes. Recent research indicates that triple therapy may provide enhanced survival advantages in particular populations, including patients with portal vein tumor thrombosis (PVTT) or those demonstrating early biomarker responses (e.g., AFP reduction >20% post-treatment) (86, 87) A multicenter retrospective study revealed that patients with BCLC stage B/C, possessing preserved liver function (Child-Pugh A) and satisfactory performance status (ECOG 0-1), experienced a significantly extended median overall survival (26.8 vs. 18.3 months, p=0.003) when treated with TACE-L-P as opposed to TACE-L, despite a higher occurrence of grade ≥3 hypertension (35% vs. 18%) (87) To alleviate negative effects, sequential administration strategies—such as commencing TACE to diminish tumor burden prior to the introduction of PD-1 inhibitors—have demonstrated potential in reducing immune-related hepatotoxicity while preserving efficacy (63). Molecular profiling, such as elevated expression of CDC20, LPCAT1, and SPP1, may identify patients predisposed to TACE resistance, therefore warranting early escalation to triple therapy. Subsequent randomized trials should further corroborate these selection criteria to enhance risk-benefit ratios (88).

The present study had several notable strengths. First, our study was an updated meta-analysis to compare the effectiveness and safety of TACE-L-P versus TACE-L for intermediate or advanced HCC. Considering the growing use of TACE-L-P and the remaining contentious regarding its effectiveness and safety, this meta-analysis provided clearer insights into the effectiveness and safety of TACE-L-P versus TACE-L for intermediate or advanced HCC and informing clinical decision-making. In addition, the heterogeneity of this meta-analysis was low, and the results were stable. Furthermore, the IPDformKM software was used to reconstruct KM curves for OS and PFS, which provide a clear and understandable representation of oncological outcomes. According to the findings of this meta-analysis, TACE-L-P should be recommended for intermediate or advanced HCC, especially for patients with BCLC stage B/C, possessing preserved liver function (Child-Pugh A) and satisfactory performance status (ECOG 0-1). Physicians must emphasize the surveillance of medication toxicity and hepatic function subsequent to the initiation of treatment, along with timely management for adverse events.

However, our study was subject to various limitations. First, as was the case with any meta-analysis, the intrinsic variability across the studies included in terms of patient baseline characteristics, disease stage, and treatment approaches might have influenced the findings. Second, since our study mostly encompassed studies conducted in China, additional research is necessary to assess the efficacy of this treatment combination in other ethnic groups and geographic populations. Third, the heterogeneity in outcomes like PFS or AEs might be caused by BCLC stage, genotype, patient age, the choice of TACE methods and chemotherapeutic drugs, but we failed to conduct further subgroup analysis due to the limitation of data. Besides, all the studies included were of a retrospective nature and resulted in low GRADE evidence grading, leading to potential bias and compromising the validity of the results. Well planned randomized controlled trials are necessary for future prospective validation of these findings. Real-world studies are also recommended to verify the observed advantages of TACE-L-P.

In conclusion, the current meta-analysis confirmed the efficacy and safety of TACE-L-P compared to TACE-L in patients with intermediate or advanced HCC. Enhancements in ORR, DCR, PFS, and OS were observed in patients with intermediate or advanced HCC who received TACE-L-P, as compared to those who received TACE-L. Furthermore, the increased effectiveness of TACE-L-P therapy was accompanied by an increase in adverse effects.

## Data Availability

The datasets presented in this study can be found in online repositories. The names of the repository/repositories and accession number(s) can be found in the article/**Supplementary Material**.

## Supplementary material

Supplementary Material 1 Comprehensive listing of the search results.

Supplementary Material 2 Supplementary Figures.

Supplementary Material 3 Survival curve reconstruction data.
