# Supplementary material for: Efficacy and safety of lenvatinib plus transarterial chemoembolization with or without programmed death-1 inhibitors in the treatment of intermediate or advanced hepatocellular carcinoma: a systematic review and meta-analysis
Source: Front Immunol. 2025 Jul 24;16:1586914. doi: 10.3389/fimmu.2025.1586914 (PMC12328301; doi:10.3389/fimmu.2025.1586914)
Supplement: Supplementary Material 1 — Comprehensive listing of the search results. [file DataSheet1.pdf]

PubMed		
No.	Query	Results
#1	"hepatocellular carcinoma"[Title/Abstract] OR "carcinomas hepatocellular"[Title/Abstract] OR "hepatocellular carcinomas"[Title/Abstract] OR (("carcinoma, hepatocellular"[MeSH Terms] OR ("Carcinoma"[All Fields] AND "Hepatocellular"[All Fields]) OR "hepatocellular carcinoma"[All Fields] OR ("Liver"[All Fields] AND "Cell"[All Fields] AND "Carcinoma"[All Fields]) OR "liver cell carcinoma"[All Fields]) AND "Adult"[Title/Abstract]) OR"adult liver cancer"[Title/Abstract] OR "adult liver cancers"[Title/Abstract] OR (("cancer s"[All Fields] OR "cancerization"[All Fields] OR "cancerized"[All Fields] OR "cancerous"[All Fields] OR "neoplasms"[MeSH Terms] OR "neoplasms"[All Fields] OR "Cancer"[All Fields] OR "Cancers"[All Fields]) AND "adult liver"[Title/Abstract]) OR (("cancer s"[All Fields] OR "cancerization"[All Fields] OR "cancerized"[All Fields] OR "cancerous"[All Fields] OR "neoplasms"[MeSH Terms] OR "neoplasms"[All Fields] OR "Cancer"[All Fields] OR "Cancers"[All Fields]) AND "adult liver"[Title/Abstract]) OR (("Liver"[MeSH Terms] OR "Liver"[All Fields] OR "livers"[All Fields] OR "liver s"[All Fields]) AND "cancers adult"[Title/Abstract]) OR "liver cell carcinoma"[Title/Abstract] OR "carcinoma liver cell"[Title/Abstract] OR (("Carcinoma"[MeSH Terms] OR "Carcinoma"[All Fields] OR "Carcinomas"[All Fields] OR "carcinoma s"[All Fields]) AND "liver cell"[Title/Abstract]) OR "cell carcinoma liver"[Title/Abstract] OR "cell carcinomas liver"[Title/Abstract] OR "liver cell carcinomas"[Title/Abstract] OR "hepatocellular carcinoma"[Title/Abstract] OR "Hepatoma"[Title/Abstract] OR "Hepatomas"[Title/Abstract]	164,848
#2	(((((((((((((lenvatinib[Title/Abstract]) OR (4-(3-chloro-4-((cyclopropylaminocarbonyl)amino)phenoxy)-7-methoxy-6-quinolinecarboxamide[Title/Abstract])) OR (Lenvima[Title/Abstract])) OR (E 7080[Title/Abstract])) OR (E-7080[Title/Abstract])) OR (ER-203492-00[Title/Abstract])) OR (E7080[Title/Abstract])) OR (E-7080 mesylate[Title/Abstract])) OR (E7080 mesylate[Title/Abstract])) OR (lenvatinib metabolite M2[Title/Abstract])) OR (4-(3-chloro-4-(((cyclopropylamino)carbonyl)amino)phenoxy)-7-hydroxy-6-quinolinecarboxamide[Title/Abstract])) OR (lenvatinib mesylate[Title/Abstract])) OR (lenvatinib methanesulfonate[Title/Abstract])) OR (N-(4-((6-carbamoyl-7-methoxyquinolin-4-yl)oxy)-2-chlorophenyl)-N'-cyclopropylurea monomethanesulfonate[Title/Abstract])) OR (lenvatinib mesilate[Title/Abstract])	3,201
#3	"transarterial chemoembolization"[Title/Abstract] OR "chemoembolization therapeutic"[Title/Abstract] OR "therapeutic chemoembolization"[Title/Abstract] OR (("chemoembolic"[All Fields] OR	11,037

	"chemoembolisation"[All Fields] OR "chemoembolisations"[All Fields] OR "chemoembolism"[All Fields] OR "Chemoembolization"[All Fields] OR "Chemoembolizations"[All Fields] OR "chemoembolized"[All Fields]) AND "Therapeutic"[Title/Abstract]) OR (("therapeutical"[All Fields] OR "therapeutically"[All Fields] OR "therapeuticals"[All Fields] OR "therapeutics"[MeSH Terms] OR "therapeutics"[All Fields] OR "Therapeutic"[All Fields]) AND "Chemoembolizations"[Title/Abstract]) OR "TACE"[Title/Abstract]	
#4	"immune checkpoint inhibitors"[Title/Abstract] OR "programmed cell death 1 inhibitor"[Title/Abstract] OR "immunotherapy therapies"[Title/Abstract] OR "Pembrolizumab"[Title/Abstract] OR "Nivolumab"[Title/Abstract] OR "Toripalimab"[Title/Abstract] OR "Tislelizumab"[Title/Abstract] OR "Camrelizumab"[Title/Abstract] OR "GLS-010"[Title/Abstract] OR "Cemiplimab"[Title/Abstract] OR "Sintilimab"[Title/Abstract] OR "Zimberelimab"[Title/Abstract] OR "Prolgolimab"[Title/Abstract] OR "Dostarlimab"[Title/Abstract]	43,366
#5	"randomized controlled trial"[Publication Type] OR "randomized controlled trials as topic"[MeSH Terms] OR "randomized controlled trials"[All Fields] OR "randomised controlled trials"[All Fields] OR "randomized controlled trials"[Title/Abstract] OR "clinical trials randomized"[Title/Abstract] OR "trials randomized clinical"[Title/Abstract] OR "controlled clinical trials randomized"[Title/Abstract]	901,494
#6	"Prospective Studies"[MeSH Terms] OR "prospective study"[Title/Abstract] OR "studies prospective"[Title/Abstract] OR "study prospective"[Title/Abstract]	769,755
#7	"Retrospective Studies"[MeSH Terms] OR "Retrospective Studies"[Title/Abstract] OR "studies retrospective"[Title/Abstract] OR "study retrospective"[Title/Abstract] OR "retrospective study"[Title/Abstract]	901,494
#8	#1AND#2AND#3 AND#4	170
#9	#5OR#6OR#7	2,861,256
#10	#8AND#9	80

Web of science		
No.	Query	Results
#1	Hepatocellular carcinoma (Topic) or Carcinomas, Hepatocellular (Topic) or Hepatocellular Carcinomas (Topic) or Liver Cell Carcinoma, Adult (Topic) or Adult Liver Cancer (Topic) not Adult Liver Cancers (Topic) or Cancer, Adult Liver (Topic) or Cancers, Adult Liver (Topic) or Liver Cancers, Adult (Topic) or Liver Cell Carcinoma (Topic) or Carcinoma, Liver Cell (Topic) or Carcinomas, Liver Cell (Topic) or Cell Carcinoma, Liver (Topic) or Cell Carcinomas, Liver (Topic) or Liver Cell Carcinomas	427,570

	(Topic) or Hepatocellular Carcinoma (Topic) or Hepatoma (Topic) or Hepatomas (Topic) and Preprint Citation Index (Exclude – Database)	
#2	lenvatinib (Topic) or 4-(3-chloro-4-((cyclopropylaminocarbonyl)amino)phenoxy)-7-methoxy-6-quinolinecarboxamide (Topic) or Lenvima (Topic) or E 7080 (Topic) or E-7080 (Topic) or ER-203492-00 (Topic) or E7080 (Topic) or E-7080 mesylate (Topic) or E7080 mesylate (Topic) or lenvatinib metabolite M2 (Topic) or 4-(3-chloro-4-(((cyclopropylamino)carbonyl)amino)phenoxy)-7-hydroxy-6-quinolinecarboxamide (Topic) or lenvatinib mesylate (Topic) or lenvatinib methanesulfonate (Topic) or N-(4-((6-carbamoyl-7-methoxyquinolin-4-yl)oxy)-2-chlorophenyl)-N'-cyclopropylurea monomethanesulfonate (Topic) or lenvatinib mesilate (Topic) and Preprint Citation Index (Exclude – Database)	5,464
#3	transarterial chemoembolization (Topic) or Chemoembolization, Therapeutic (Topic) or Therapeutic Chemoembolization (Topic) or Chemoembolizations, Therapeutic (Topic) or Therapeutic Chemoembolizations (Topic) or TACE (Topic) and Preprint Citation Index (Exclude – Database)	22,664
#4	immune checkpoint inhibitors (Topic) or programmed cell death 1 inhibitor (Topic) or immunotherapy therapies (Topic) or Pembrolizumab (Topic) or Nivolumab (Topic) or Toripalimab (Topic) or Tislelizumab (Topic) or Camrelizumab (Topic) or GLS-010 (Topic) or Cemiplimab (Topic) or Sintilimab (Topic) or Zimberelimab (Topic) or Prolgolimab (Topic) or Dostarlimab (Topic) and Preprint Citation Index (Exclude – Database)	352,534
#5	Randomized Controlled Trials (Topic) or Clinical Trials, Randomized (Topic) or Trials, Randomized Clinical (Topic) or Controlled Clinical Trials, Randomized (Topic) and Preprint Citation Index (Exclude – Database)	1,096,014
#6	Prospective Study (Topic) or Studies, Prospective (Topic) or Study, Prospective (Topic) and Preprint Citation Index (Exclude – Database)	1,281,712
#7	Retrospective Studies (Topic) or Studies, Retrospective (Topic) or Study, Retrospective (Topic) or Retrospective Study (Topic) and Preprint Citation Index (Exclude – Database)	1,691,420
#8	#1AND#2AND#3 AND#4	376
#9	#5OR#6OR#7	3,775,628
#10	#8AND#9	159

Embase		
#1	'carcinomas, hepatocellular':ti,ab,kw OR 'hepatocellular carcinomas':ti,ab,kw OR 'liver cell carcinoma, adult':ti,ab,kw OR 'adult liver cancer':ti,ab,kw OR 'adult liver cancers':ti,ab,kw OR 'cancer, adult liver':ti,ab,kw OR 'cancers, adult liver':ti,ab,kw OR 'liver cancers, adult':ti,ab,kw OR 'liver cell carcinoma':ti,ab,kw OR 'carcinoma, liver cell':ti,ab,kw OR 'carcinomas, liver cell':ti,ab,kw OR 'cell carcinoma, liver':ti,ab,kw OR 'cell carcinomas, liver':ti,ab,kw OR 'liver cell carcinomas':ti,ab,kw OR 'hepatocellular carcinoma':ti,ab,kw OR 'hepatoma':ti,ab,kw OR 'hepatomas'	227315
#2	'lenvatinib'/exp OR 'lenvatinib' OR 'lenvatinib'/exp OR lenvatinib OR (4-:ti,ab,kw AND '3 chloro 4':ti,ab,kw AND 'n cyclopropylureido':ti,ab,kw AND phenoxy:ti,ab,kw AND '7 methoxyquinoline 6 carboxamide':ti,ab,kw) OR 'e 7080':ti,ab,kw OR e7080:ti,ab,kw OR 'e7080 mesylate':ti,ab,kw OR 'lenvatinib metabolite m2':ti,ab,kw OR (4-:ti,ab,kw AND '3 chloro 4':ti,ab,kw AND cyclopropylamino:ti,ab,kw AND carbonyl:ti,ab,kw AND amino:ti,ab,kw AND phenoxy:ti,ab,kw AND '7 hydroxy 6 quinolinecarboxamide':ti,ab,kw) OR 'lenvatinib mesylate':ti,ab,kw OR 'lenvatinib methanesulfonate':ti,ab,kw OR (n-:ti,ab,kw AND 4-:ti,ab,kw AND '6 carbamoyl 7 methoxyquinolin 4 yl':ti,ab,kw AND oxy:ti,ab,kw AND '2 chlorophenyl':ti,ab,kw AND '-n-cyclopropylurea monomethanesulfonate':ti,ab,kw) OR 'lenvatinib mesilate':ti,ab,kw	10293
#3	'transarterial chemoembolization'/exp OR 'transarterial chemoembolization' OR (transarterial AND ('chemoembolization'/exp OR chemoembolization)) OR 'chemoembolization, therapeutic':ti,ab,kw OR 'therapeutic chemoembolization':ti,ab,kw OR 'chemoembolizations, therapeutic':ti,ab,kw OR 'therapeutic chemoembolizations':ti,ab,kw OR 'tace':ti,ab,kw	19536
#4	'immune checkpoint inhibitors'/exp OR 'immune checkpoint inhibitors' OR (('immune'/exp OR immune) AND ('checkpoint'/exp OR checkpoint) AND ('inhibitors'/exp OR inhibitors)) OR 'programmed cell death 1 inhibitor':ab,ti OR 'immunotherapy therapies':ab,ti OR pembrolizumab:ab,ti OR nivolumab:ab,ti OR toripalimab:ab,ti OR tislelizumab:ab,ti OR camrelizumab:ab,ti OR 'gls 010':ab,ti OR cemiplimab:ab,ti OR sintilimab:ab,ti OR zimberelimab:ab,ti OR prolgolimab:ab,ti OR dostarlimab:ab,ti	104447
#5	'randomized controlled trials'/exp OR 'randomized controlled trials' OR (randomized AND controlled AND trials) OR 'clinical trials, randomized':ti,ab,kw OR 'trials, randomized clinical':ti,ab,kw OR	520288

	'controlled clinical trials, randomized':ti,ab,kw	
#6	'prospective study'/exp OR 'prospective study' OR (prospective AND ('study'/exp OR study)) OR 'studies, prospective':ti,ab,kw OR 'study, prospective':ti,ab,kw	1475578
#7	'retrospective studies'/exp OR 'retrospective studies' OR (retrospective AND ('studies'/exp OR studies)) OR 'studies, retrospective':ti,ab,kw OR 'study, retrospective':ti,ab,kw OR 'retrospective study':ti,ab,kw	1936890
#8	#1AND#2AND#3 AND#4	284
#9	#5OR#6OR#7	3721950
#10	#8AND#9	426

cochrane library		
No.	Query	Results
#1	(Hepatocellular carcinoma OR Carcinomas, Hepatocellular OR Hepatocellular Carcinomas OR Liver Cell Carcinoma, Adult OR Adult Liver Cancer OR Adult Liver Cancers OR Cancer, Adult Liver OR Cancers, Adult Liver OR Liver Cancers, Adult OR Liver Cell Carcinoma OR Carcinoma, Liver Cell OR Carcinomas, Liver Cell OR Cell Carcinoma, Liver OR Cell Carcinomas, Liver OR Liver Cell Carcinomas OR Hepatocellular Carcinoma OR Hepatoma OR Hepatomas):ab,ti,kw	11966
#2	(lenvatinib OR Lenvima OR E 7080 OR E-7080 OR E7080 OR E-7080 mesylate OR E7080 mesylate OR lenvatinib metabolite M2 OR lenvatinib mesylate OR lenvatinib methanesulfonate OR lenvatinib mesilate):ab,ti,kw	769
#3	(transarterial chemoembolization OR Chemoembolization, Therapeutic OR Therapeutic Chemoembolization OR Chemoembolizations, Therapeutic OR Therapeutic Chemoembolizations OR TACE):ab,ti,kw	1094
#4	(immune checkpoint inhibitors OR programmed cell death 1 inhibitor OR immunotherapy therapies OR PD-1 inhibitor OR Pembrolizumab OR Nivolumab OR Toripalimab OR Camrelizumab OR GLS-010 OR Cemiplimab OR Sintilimab OR Zimberelimab OR Prolgolimab OR Dostarlimab):ab,ti,kw	7498
#5	(Randomized Controlled Trials OR Clinical Trials, Randomized OR Trials, Randomized Clinical OR Controlled Clinical Trials, Randomized):ab,ti,kw	213600
#6	(Prospective Study OR Studies, Prospective OR Study, Prospective):ab,ti,kw	253385
#7	(Retrospective Studies OR Studies, Retrospective OR Study, Retrospective OR Retrospective Study):ab,ti,kw	40228
#8	#1AND#2AND#3 AND#4	24
#9	#5OR#6OR#7	461634

#10	#8AND#9	11

Additional manual record

(1) Ando, Y.; Kawaoka, T.; Amioka, K.; Naruto, K.; Ogawa, Y.; Yoshikawa, Y.; Kikukawa, C.; Kosaka, Y.; Uchikawa, S.; Morio, K.; et al. Efficacy and Safety of Lenvatinib-Transcatheter Arterial Chemoembolization Sequential Therapy for Patients with Intermediate-Stage Hepatocellular Carcinoma. *Oncology* **2021**, *99* (8), 507-517. DOI: 10.1159/000515865 From NLM.

(2) Chen, R.; Li, Y.; Song, K.; Li, L.; Shen, C.; Ma, P.; Wang, Z. Efficacy and safety of transarterial chemoembolization-lenvatinib sequential therapy for the treatment of hepatocellular carcinoma with portal vein tumor thrombus: a retrospective study. *J Gastrointest Oncol* **2022**, *13* (2), 780-786. DOI: 10.21037/jgo-22-239 From NLM.

(3) He, C.; Ge, N.; Wang, X.; Li, H.; Chen, S.; Yang, Y. Conversion Therapy of Large Unresectable Hepatocellular Carcinoma With Ipsilateral Portal Vein Tumor Thrombus Using Portal Vein Embolization Plus Transcatheter Arterial Chemoembolization. *Front Oncol* **2022**, *12*, 923566. DOI: 10.3389/fonc.2022.923566 From NLM.

(4) Kudo, M.; Ueshima, K.; Saeki, I.; Ishikawa, T.; Inaba, Y.; Morimoto, N.; Aikata, H.; Tanabe, N.; Wada, Y.; Kondo, Y.; et al. A Phase 2, Prospective, Multicenter, Single-Arm Trial of Transarterial Chemoembolization Therapy in Combination Strategy with Lenvatinib in Patients with Unresectable Intermediate-Stage Hepatocellular Carcinoma: TACTICS-L Trial. *Liver Cancer* **2024**, *13* (1), 99-112. DOI: 10.1159/000531377 From NLM.

(5) Kuroda, H.; Oikawa, T.; Ninomiya, M.; Fujita, M.; Abe, K.; Okumoto, K.; Katsumi, T.; Sato, W.; Igarashi, G.; Iino, C.; et al. Objective Response by mRECIST to Initial Lenvatinib Therapy Is an Independent Factor Contributing to Deep Response in Hepatocellular Carcinoma Treated with Lenvatinib-Transcatheter Arterial Chemoembolization Sequential Therapy. *Liver Cancer* **2022**, *11* (4), 383-396. DOI: 10.1159/000522424 From NLM.

(6) Lin, K.; Wei, F.; Huang, Q.; Lai, Z.; Zhang, J.; Chen, Q.; Jiang, Y.; Kong, J.; Tang, S.; Lin, J.; et al. Postoperative Adjuvant Transarterial Chemoembolization Plus Tyrosine Kinase Inhibitor for Hepatocellular Carcinoma: a Multicentre Retrospective Study. *J Hepatocell Carcinoma* **2022**, *9*, 127-140. DOI: 10.2147/jhc.S352480 From NLM.

(7) Liu, J.; Yan, S.; Zhang, G.; Yang, L.; Wei, S.; Yi, P. A retrospective study of transarterial chemoembolization (TACE) combined with lenvatinib compared with TACE monotherapy for BCLC B2 stage hepatocellular carcinoma. *Oncol Lett* **2023**, *26* (6), 507. DOI: 10.3892/ol.2023.14094 From NLM.
