# Supplementary material for: Efficacy and safety of lenvatinib plus transarterial chemoembolization with or without programmed death-1 inhibitors in the treatment of intermediate or advanced hepatocellular carcinoma: a systematic review and meta-analysis
Source: Front Immunol. 2025 Jul 24;16:1586914. doi: 10.3389/fimmu.2025.1586914 (PMC12328301; doi:10.3389/fimmu.2025.1586914)

**Figure S1.** Sensitive analysis for ORR

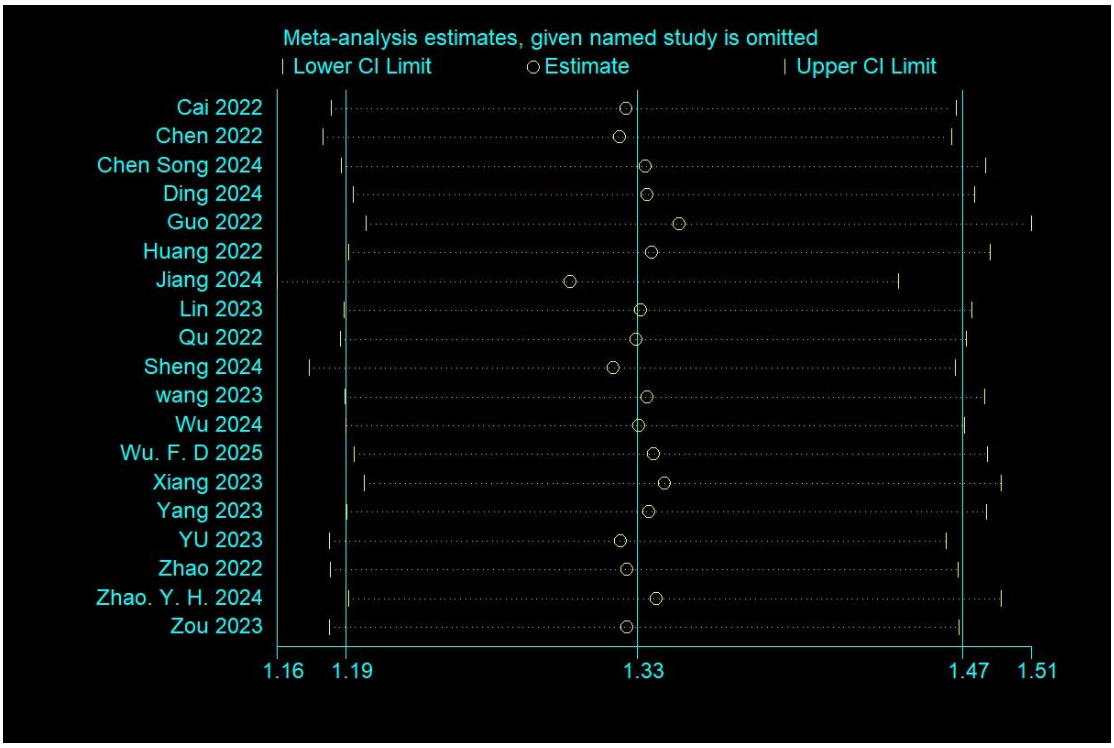


**Figure S2.** Sensitive analysis for DCR

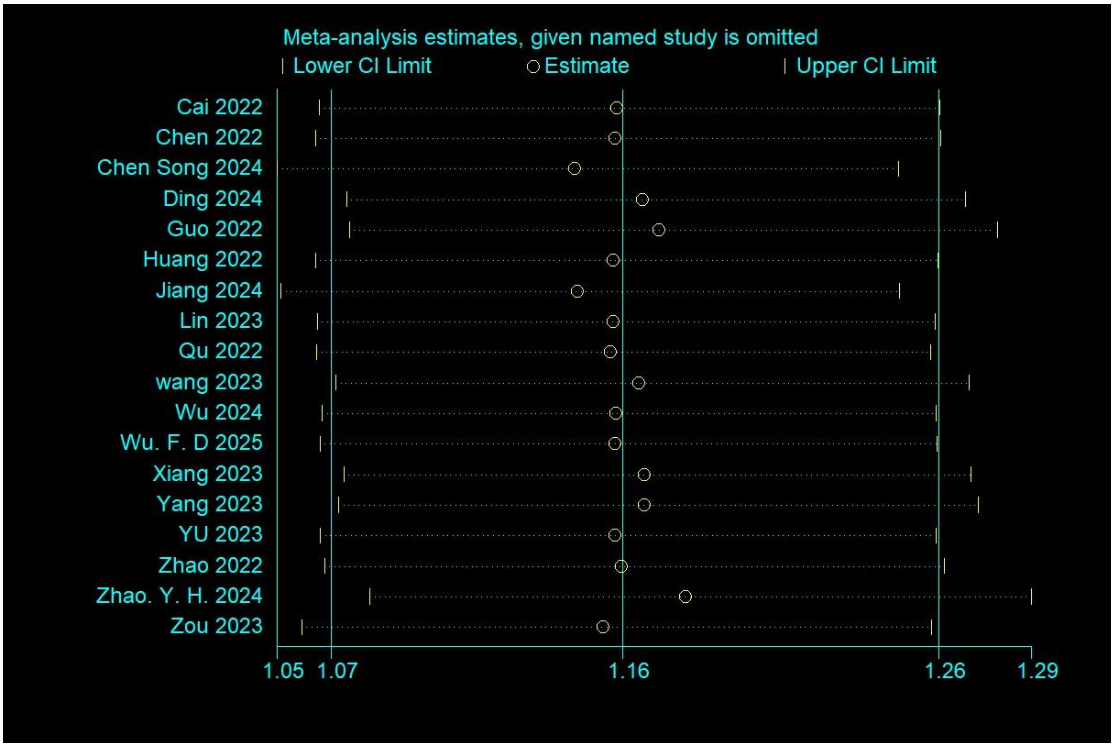


**Figure S3.** Sensitive analysis for PFS

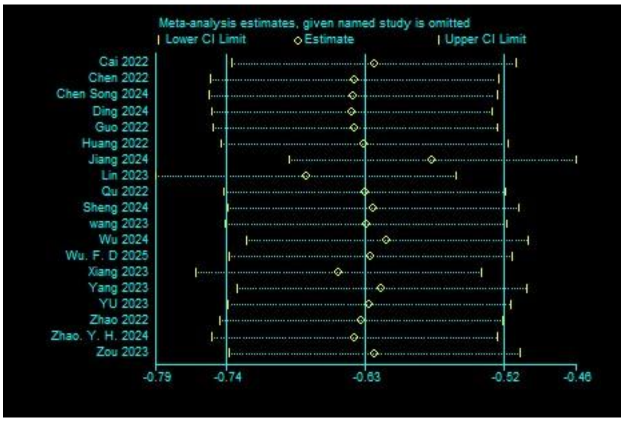


**Figure S4.** Sensitive analysis for OS

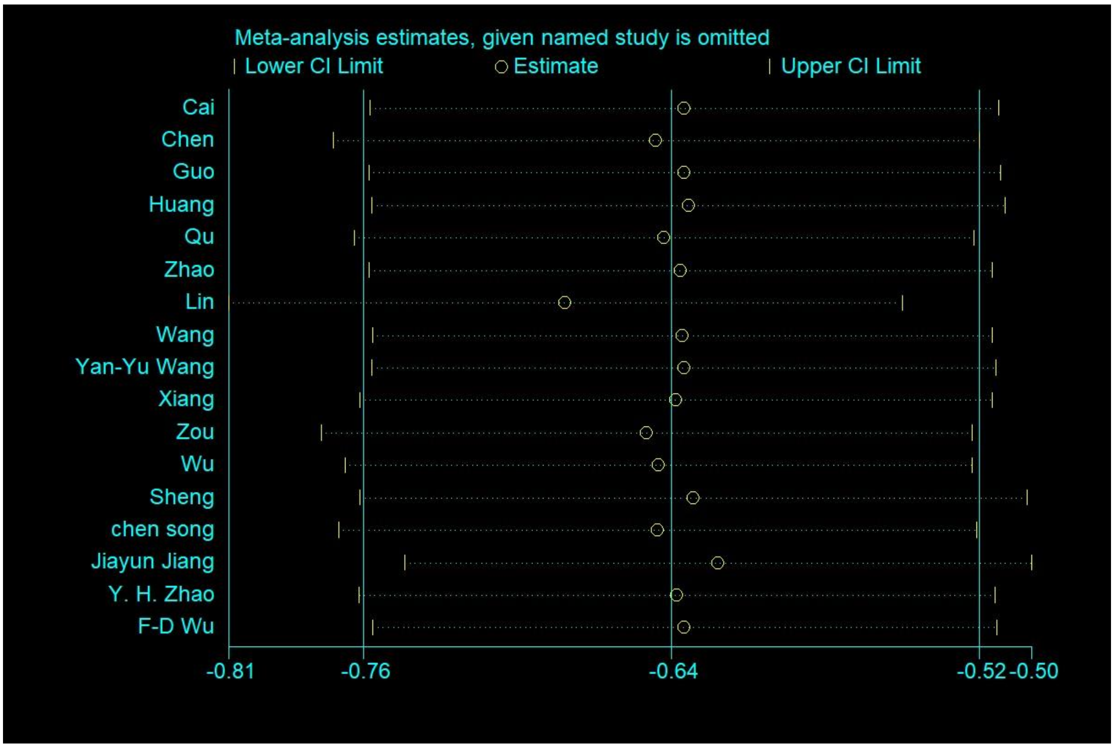


**Figure S5.**Sensitive analysis for Grade≥ 3 AEs rate

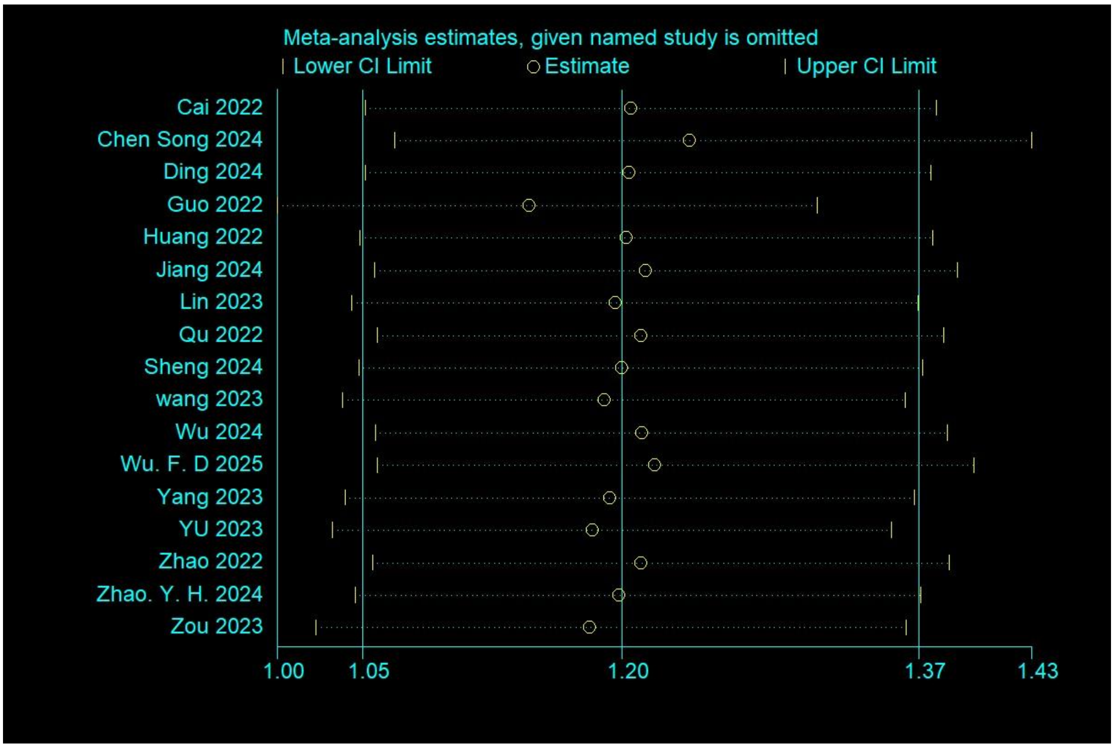


**Figure S6.** funnel plot for ORR

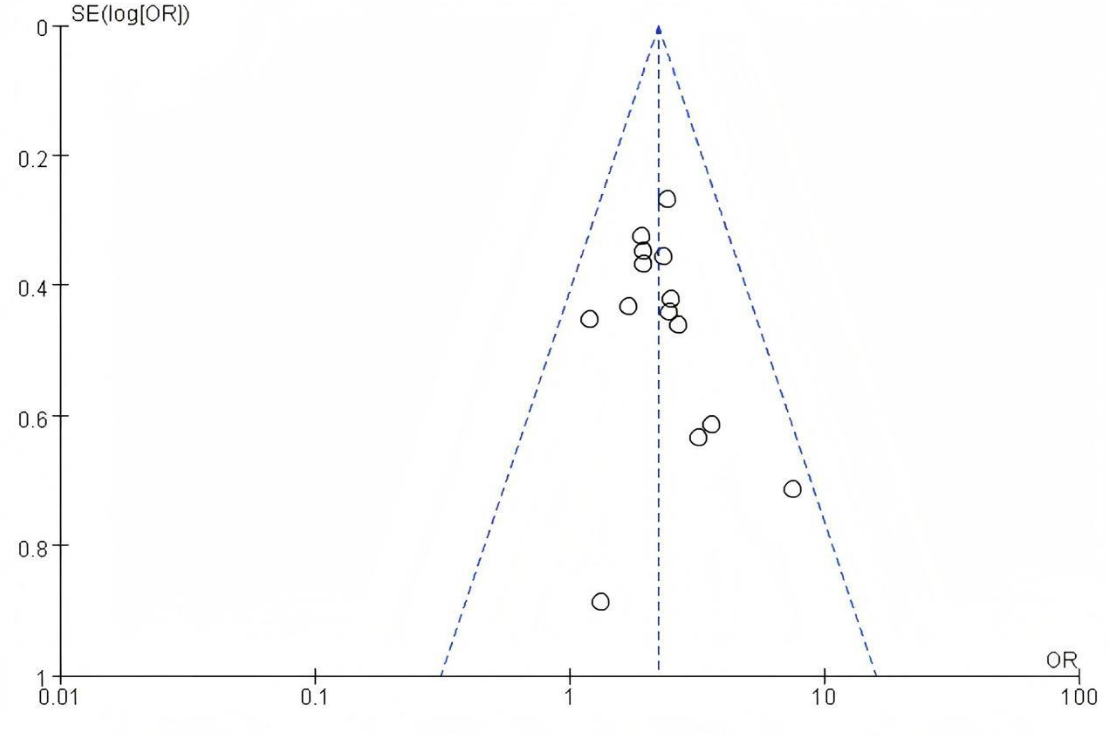


**Figure S7.** funnel plot for DCR

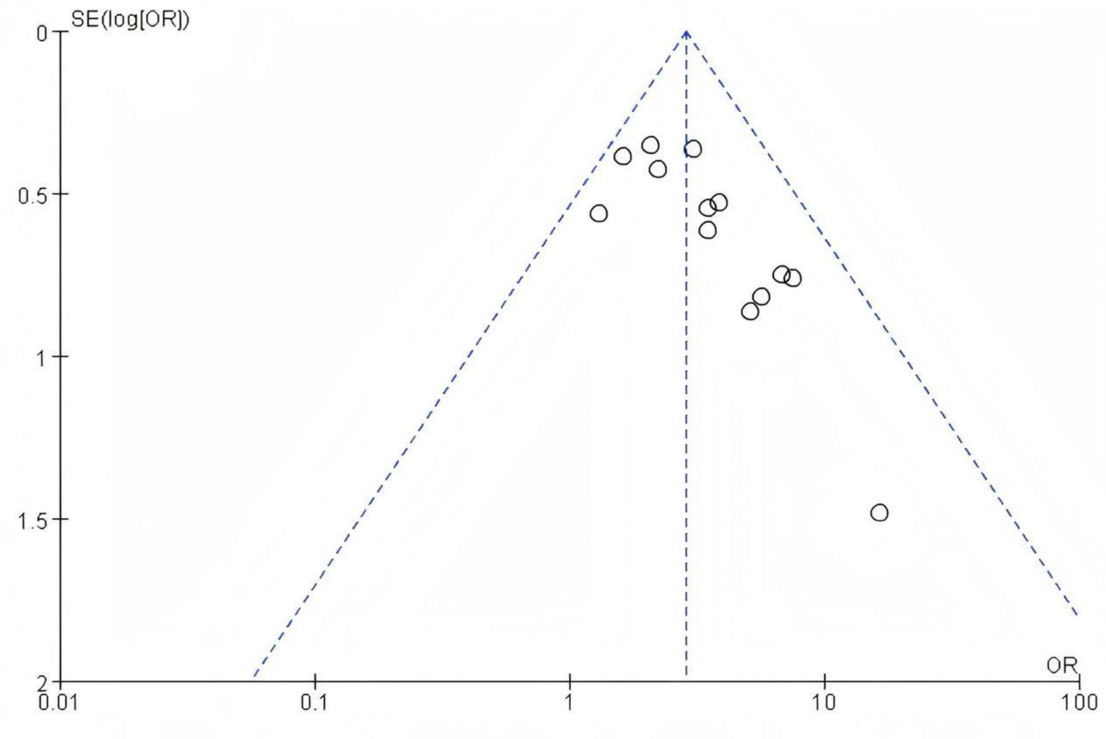


**Figure S8.** funnel plot for PFS

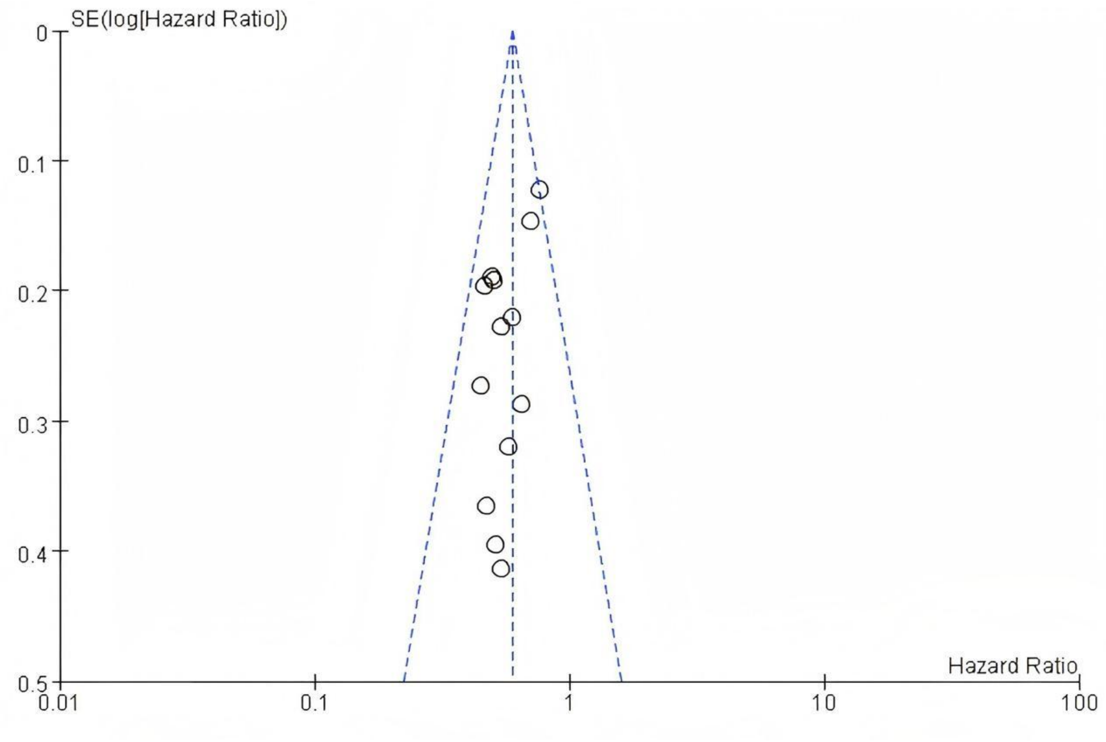


**Figure S9.** funnel plot for OS

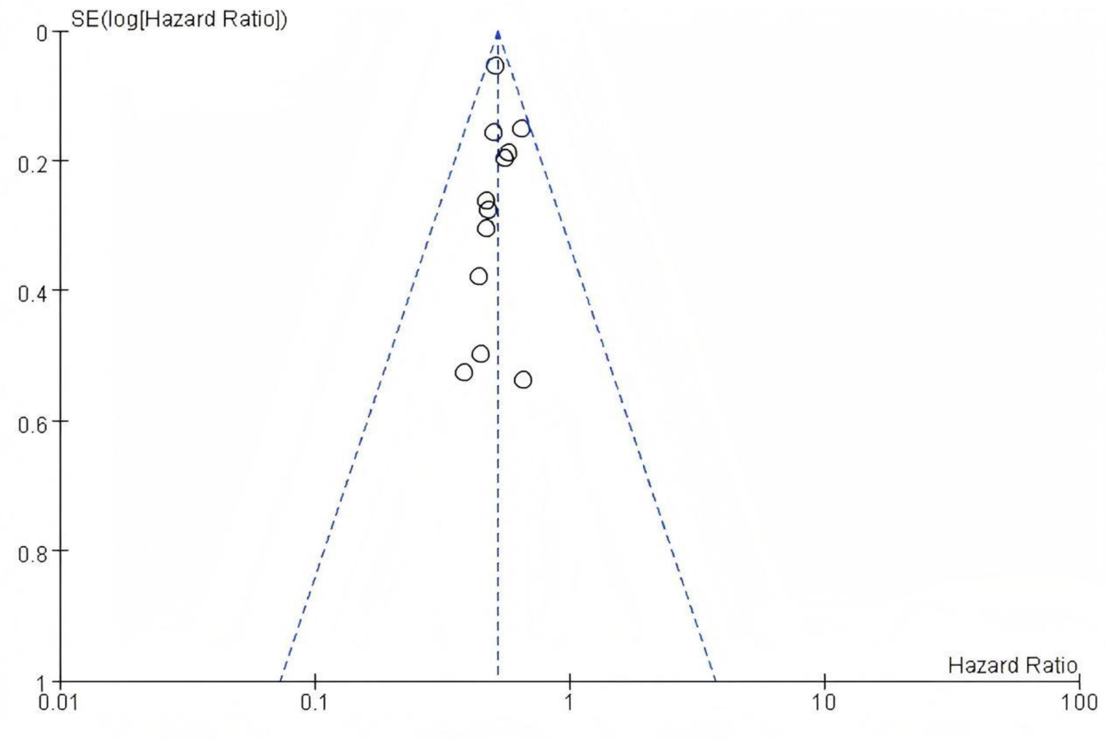


**Figure S10.** funnel plot for Grade≥ 3 AEs rate

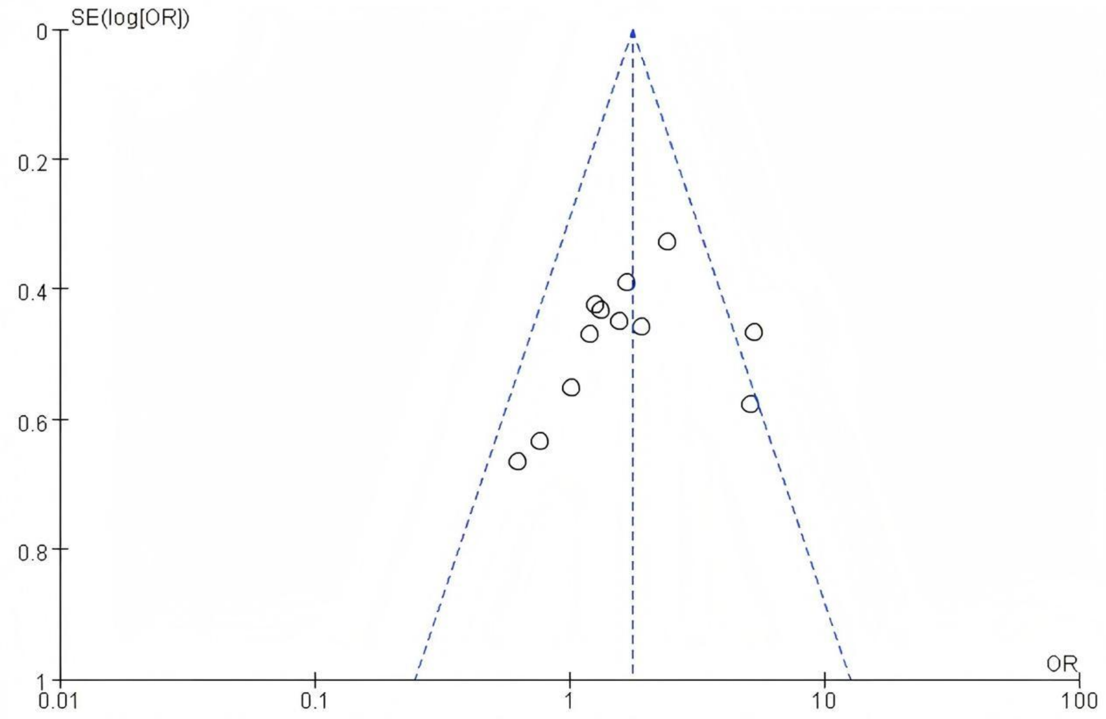


**Figure S11.**Subgroup analysis regarding hypertension

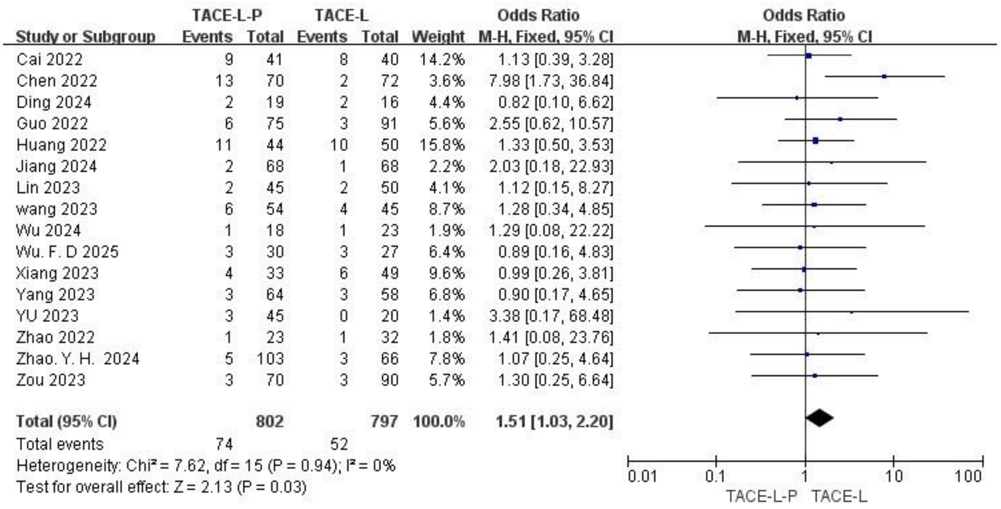


**Figure S12.**Subgroup analysis regarding rash

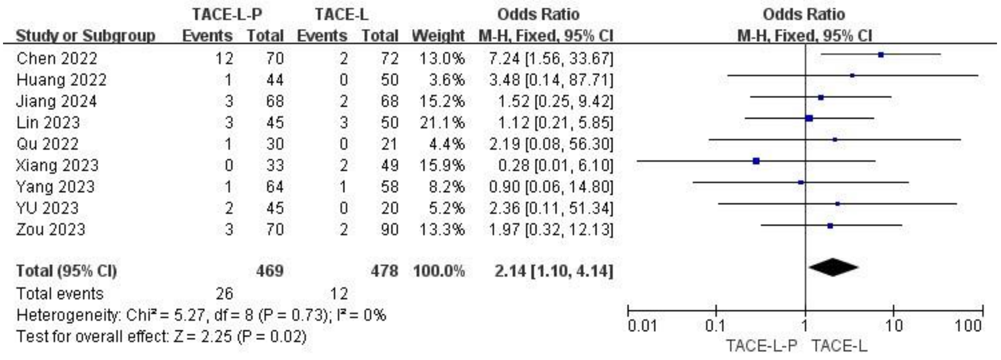


**Figure S13.**Subgroup analysis regarding Decreased appetite

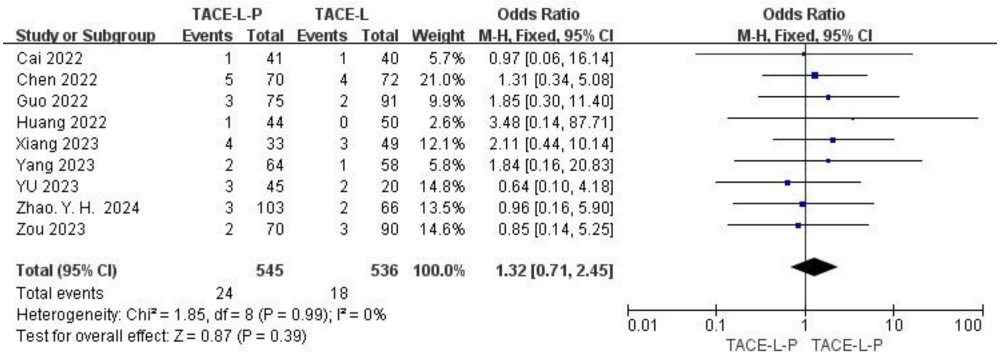


**Figure S14.**Subgroup analysis regarding Elevated AST

| Study or Subgroup | TACE-L-P Events | TACE-L-P Total | TACE-L Events | TACE-L Total | Weight | Odds Ratio M-H, Fixed, 95% CI |
|---|---|---|---|---|---|---|
| Cai 2022 | 0 | 41 | 1 | 40 | 2.9% | 0.32 [0.01, 8.02] |
| Chen 2022 | 17 | 70 | 11 | 72 | 15.7% | 1.78 [0.77, 4.13] |
| Chen Song 2024 | 21 | 66 | 15 | 45 | 23.2% | 0.93 [0.42, 2.09] |
| Guo 2022 | 1 | 75 | 0 | 91 | 0.8% | 3.68 [0.15, 91.78] |
| Jiang 2024 | 15 | 68 | 18 | 68 | 26.8% | 0.79 [0.36, 1.73] |
| Lin 2023 | 2 | 45 | 1 | 50 | 1.7% | 2.28 [0.20, 26.02] |
| Qu 2022 | 2 | 30 | 4 | 21 | 8.4% | 0.30 [0.05, 1.84] |
| Sheng 2024 | 1 | 113 | 2 | 128 | 3.5% | 0.56 [0.05, 6.29] |
| wang 2023 | 2 | 54 | 1 | 45 | 2.0% | 1.69 [0.15, 19.30] |
| Wu 2024 | 2 | 18 | 3 | 23 | 4.5% | 0.83 [0.12, 5.61] |
| YU 2023 | 3 | 45 | 0 | 20 | 1.2% | 3.38 [0.17, 68.48] |
| Zhao 2022 | 4 | 23 | 7 | 32 | 9.2% | 0.75 [0.19, 2.95] |
| **Total (95% CI)** | | **648** | | **635** | **100.0%** | **1.01 [0.69, 1.48]** |
| Total events | 70 | | 63 | | | |

Heterogeneity: Chi² = 6.65, df = 11 (P = 0.83); I² = 0%
Test for overall effect: Z = 0.07 (P = 0.95)

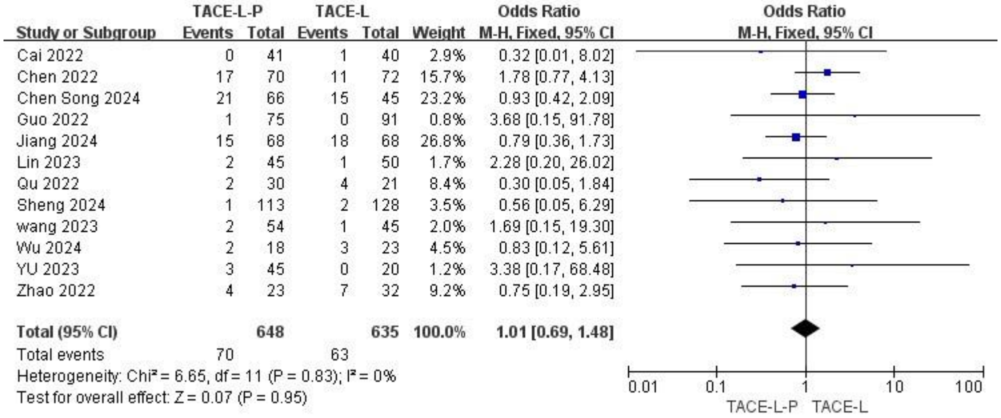


**Figure S15.**Subgroup analysis regarding Elevated ALT

| Study or Subgroup | TACE-L-P Events | TACE-L-P Total | TACE-L Events | TACE-L Total | Weight | Odds Ratio M-H, Fixed, 95% CI |
|---|---|---|---|---|---|---|
| Cai 2022 | 1 | 41 | 0 | 40 | 1.0% | 3.00 [0.12, 75.85] |
| Chen 2022 | 16 | 70 | 12 | 72 | 19.6% | 1.48 [0.64, 3.41] |
| Chen Song 2024 | 21 | 66 | 15 | 45 | 26.1% | 0.93 [0.42, 2.09] |
| Guo 2022 | 1 | 75 | 0 | 91 | 1.0% | 3.68 [0.15, 91.78] |
| Jiang 2024 | 9 | 68 | 10 | 68 | 18.6% | 0.88 [0.34, 2.34] |
| Lin 2023 | 2 | 45 | 1 | 50 | 1.9% | 2.28 [0.20, 26.02] |
| Qu 2022 | 1 | 75 | 0 | 91 | 1.0% | 3.68 [0.15, 91.78] |
| Sheng 2024 | 4 | 113 | 5 | 128 | 9.7% | 0.90 [0.24, 3.45] |
| wang 2023 | 5 | 54 | 3 | 45 | 6.4% | 1.43 [0.32, 6.34] |
| Wu 2024 | 2 | 18 | 3 | 23 | 5.0% | 0.83 [0.12, 5.61] |
| YU 2023 | 2 | 45 | 0 | 20 | 1.4% | 2.36 [0.11, 51.34] |
| Zhao 2022 | 5 | 23 | 6 | 32 | 8.4% | 1.20 [0.32, 4.55] |
| **Total (95% CI)** | | **693** | | **705** | **100.0%** | **1.20 [0.81, 1.77]** |
| Total events | 69 | | 55 | | | |

Heterogeneity: Chi² = 3.06, df = 11 (P = 0.99); I² = 0%
Test for overall effect: Z = 0.91 (P = 0.36)

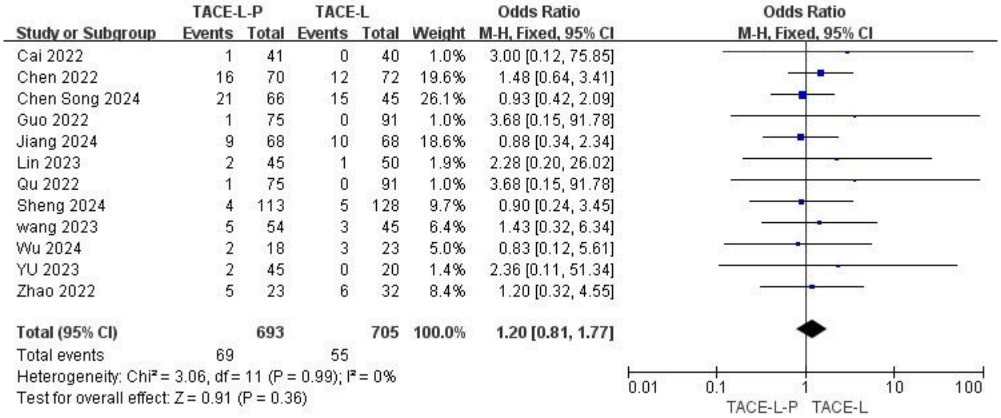


**Figure S16.**Subgroup analysis regarding Fatigue

| Study or Subgroup | TACE-L-P Events | TACE-L-P Total | TACE-L Events | TACE-L Total | Weight | Odds Ratio M-H, Fixed, 95% CI |
|---|---|---|---|---|---|---|
| Cai 2022 | 1 | 41 | 0 | 40 | 2.5% | 3.00 [0.12, 75.85] |
| Chen 2022 | 7 | 70 | 2 | 72 | 9.0% | 3.89 [0.78, 19.41] |
| Guo 2022 | 4 | 75 | 2 | 91 | 8.7% | 2.51 [0.45, 14.08] |
| Jiang 2024 | 1 | 68 | 1 | 68 | 5.0% | 1.00 [0.06, 16.32] |
| Lin 2023 | 2 | 45 | 2 | 50 | 9.2% | 1.12 [0.15, 8.27] |
| Wu. F. D 2025 | 3 | 30 | 2 | 27 | 9.6% | 1.39 [0.21, 9.01] |
| Xiang 2023 | 3 | 33 | 2 | 49 | 7.4% | 2.35 [0.37, 14.90] |
| Yang 2023 | 3 | 64 | 2 | 58 | 10.2% | 1.38 [0.22, 8.55] |
| YU 2023 | 3 | 45 | 1 | 20 | 6.6% | 1.36 [0.13, 13.91] |
| Zhao 2022 | 1 | 23 | 1 | 32 | 4.1% | 1.41 [0.08, 23.76] |
| Zhao. Y. H. 2024 | 1 | 103 | 0 | 66 | 3.0% | 1.95 [0.08, 48.49] |
| Zou 2023 | 5 | 70 | 6 | 90 | 24.8% | 1.08 [0.31, 3.69] |
| **Total (95% CI)** | | **667** | | **663** | **100.0%** | **1.72 [0.99, 2.98]** |
| Total events | 34 | | 21 | | | |

Heterogeneity: Chi² = 2.45, df = 11 (P = 1.00); I² = 0%
Test for overall effect: Z = 1.92 (P = 0.06)

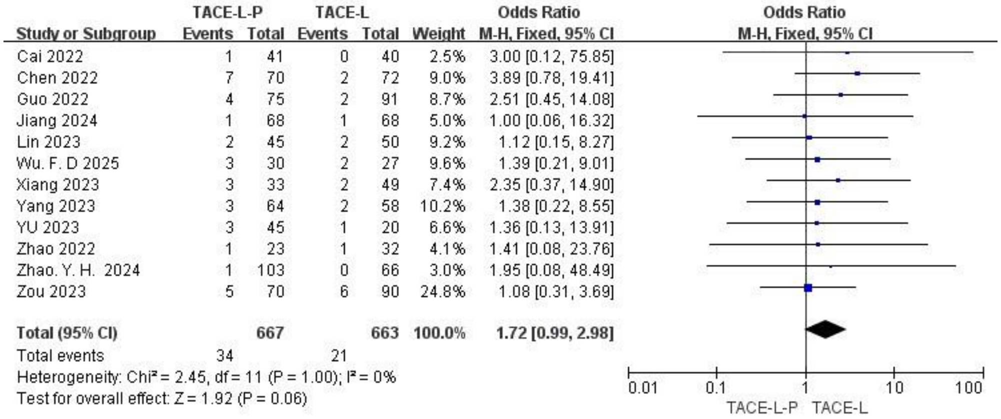


**Figure S17.**Subgroup analysis regarding Diarrhea

| Study or Subgroup | TACE-L-P Events | TACE-L-P Total | TACE-L Events | TACE-L Total | Weight | Odds Ratio M-H, Fixed, 95% CI |
|---|---|---|---|---|---|---|
| Chen 2022 | 5 | 70 | 3 | 72 | 10.5% | 1.77 [0.41, 7.70] |
| Chen Song 2024 | 8 | 66 | 5 | 45 | 20.0% | 1.10 [0.34, 3.62] |
| Guo 2022 | 4 | 75 | 0 | 91 | 1.6% | 11.52 [0.61, 217.44] |
| Huang 2022 | 2 | 44 | 2 | 50 | 6.9% | 1.14 [0.15, 8.47] |
| Jiang 2024 | 1 | 68 | 1 | 68 | 3.8% | 1.00 [0.06, 16.32] |
| Sheng 2024 | 6 | 113 | 6 | 128 | 20.4% | 1.14 [0.36, 3.64] |
| Wu. F. D 2025 | 2 | 30 | 2 | 27 | 7.5% | 0.89 [0.12, 6.82] |
| Xiang 2023 | 0 | 33 | 1 | 49 | 4.6% | 0.48 [0.02, 12.21] |
| Yang 2023 | 1 | 64 | 1 | 58 | 4.0% | 0.90 [0.06, 14.80] |
| YU 2023 | 5 | 45 | 1 | 20 | 4.7% | 2.38 [0.26, 21.77] |
| Zhao 2022 | 1 | 23 | 1 | 32 | 3.1% | 1.41 [0.08, 23.76] |
| Zou 2023 | 3 | 70 | 4 | 90 | 12.8% | 0.96 [0.21, 4.45] |
| **Total (95% CI)** | | **701** | | **730** | **100.0%** | **1.35 [0.81, 2.24]** |
| Total events | 38 | | 27 | | | |

Heterogeneity: Chi² = 3.50, df = 11 (P = 0.98); I² = 0%
Test for overall effect: Z = 1.16 (P = 0.25)

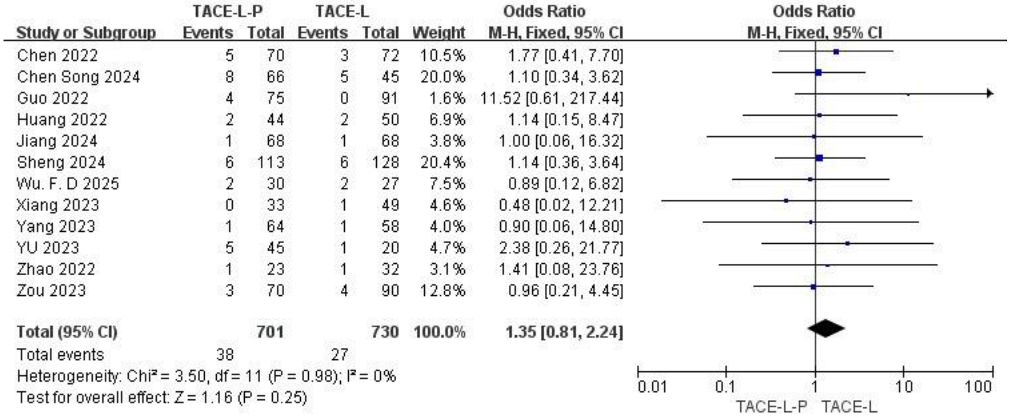


**Figure S18.**Subgroup analysis regarding Abdominal pain

| Study or Subgroup | TACE-L-P Events | TACE-L-P Total | TACE-L Events | TACE-L Total | Weight | Odds Ratio M-H, Fixed, 95% CI |
|---|---|---|---|---|---|---|
| Cai 2022 | 0 | 41 | 1 | 40 | 7.1% | 0.32 [0.01, 8.02] |
| Chen Song 2024 | 5 | 66 | 4 | 45 | 20.8% | 0.84 [0.21, 3.32] |
| Guo 2022 | 1 | 75 | 0 | 91 | 2.1% | 3.68 [0.15, 91.78] |
| Huang 2022 | 1 | 44 | 1 | 50 | 4.3% | 1.14 [0.07, 18.77] |
| Qu 2022 | 1 | 30 | 1 | 21 | 5.4% | 0.69 [0.04, 11.68] |
| Sheng 2024 | 2 | 113 | 1 | 128 | 4.4% | 2.29 [0.20, 25.58] |
| Wu. F. D 2025 | 4 | 30 | 3 | 27 | 12.9% | 1.23 [0.25, 6.07] |
| Xiang 2023 | 5 | 33 | 8 | 49 | 25.8% | 0.92 [0.27, 3.09] |
| YU 2023 | 2 | 45 | 1 | 20 | 6.3% | 0.88 [0.08, 10.35] |
| Zhao. Y. H. 2024 | 5 | 103 | 2 | 66 | 11.0% | 1.63 [0.31, 8.67] |
| **Total (95% CI)** | | **580** | | **537** | **100.0%** | **1.09 [0.60, 1.97]** |
| Total events | 26 | | 22 | | | |

Heterogeneity: Chi² = 2.07, df = 9 (P = 0.99); I² = 0%
Test for overall effect: Z = 0.29 (P = 0.77)

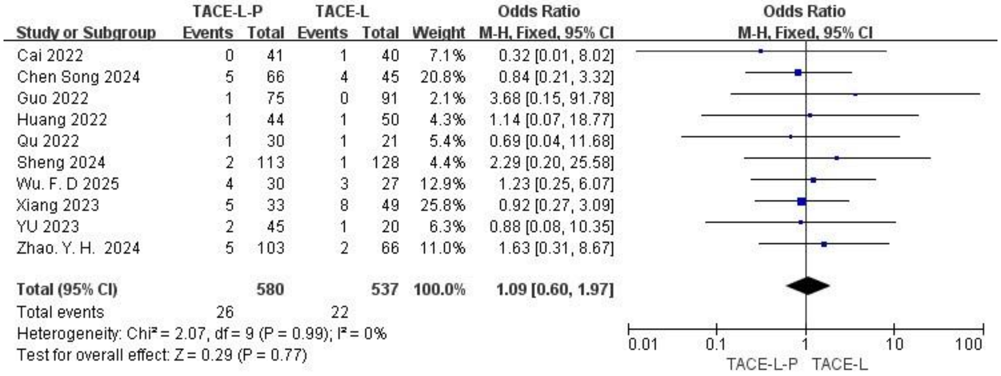


**Figure S19.**Subgroup analysis regarding Hand-foot syndrome

| Study or Subgroup | TACE-L-P Events | TACE-L-P Total | TACE-L Events | TACE-L Total | Weight | Odds Ratio M-H, Fixed, 95% CI |
|---|---|---|---|---|---|---|
| Chen Song 2024 | 4 | 66 | 3 | 45 | 13.4% | 0.90 [0.19, 4.24] |
| Ding 2024 | 4 | 19 | 3 | 16 | 10.3% | 1.16 [0.22, 6.15] |
| Guo 2022 | 1 | 75 | 0 | 91 | 1.8% | 3.68 [0.15, 91.78] |
| Huang 2022 | 0 | 44 | 1 | 50 | 5.5% | 0.37 [0.01, 9.34] |
| Jiang 2024 | 2 | 68 | 3 | 68 | 11.6% | 0.66 [0.11, 4.06] |
| Lin 2023 | 4 | 45 | 3 | 50 | 10.3% | 1.53 [0.32, 7.23] |
| Sheng 2024 | 6 | 113 | 3 | 128 | 10.6% | 2.34 [0.57, 9.57] |
| wang 2023 | 2 | 54 | 1 | 45 | 4.2% | 1.69 [0.15, 19.30] |
| Wu. F. D 2025 | 3 | 30 | 1 | 27 | 3.8% | 2.89 [0.28, 29.58] |
| Yang 2023 | 4 | 64 | 2 | 58 | 7.8% | 1.87 [0.33, 10.59] |
| Zhao. Y. H. 2024 | 2 | 103 | 1 | 66 | 4.8% | 1.29 [0.11, 14.48] |
| Zou 2023 | 6 | 70 | 5 | 90 | 15.9% | 1.59 [0.47, 5.45] |
| **Total (95% CI)** | | **751** | | **734** | **100.0%** | **1.45 [0.87, 2.41]** |
| Total events | 38 | | 26 | | | |

Heterogeneity: Chi² = 3.08, df = 11 (P = 0.99); I² = 0%
Test for overall effect: Z = 1.43 (P = 0.15)

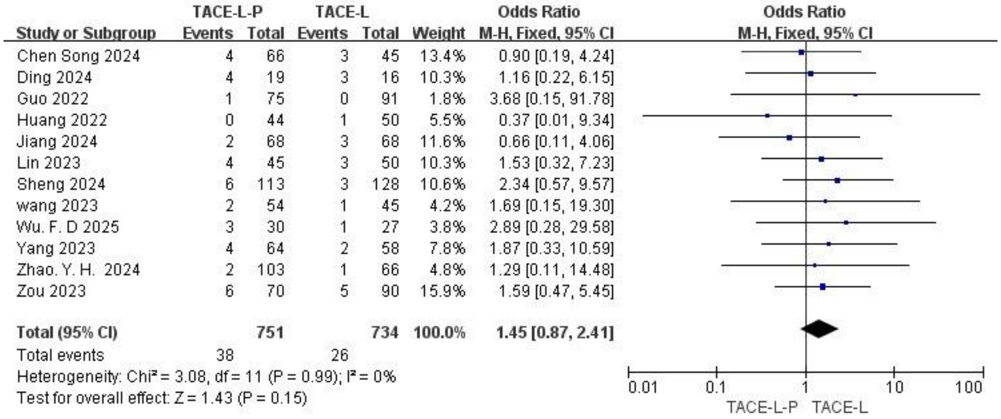


**Figure S20.**Subgroup analysis regarding Thrombocytopenia

| Study or Subgroup | TACE-L-P Events | TACE-L-P Total | TACE-L Events | TACE-L Total | Weight | Odds Ratio M-H, Fixed, 95% CI |
|---|---|---|---|---|---|---|
| Cai 2022 | 1 | 41 | 2 | 40 | 11.0% | 0.47 [0.04, 5.46] |
| Chen 2022 | 5 | 70 | 3 | 72 | 15.4% | 1.77 [0.41, 7.70] |
| Chen Song 2024 | 5 | 66 | 3 | 45 | 18.4% | 1.15 [0.26, 5.06] |
| wang 2023 | 3 | 54 | 1 | 45 | 5.8% | 2.59 [0.26, 25.79] |
| Wu 2024 | 0 | 18 | 1 | 23 | 7.2% | 0.41 [0.02, 10.55] |
| Wu. F. D 2025 | 1 | 30 | 1 | 27 | 5.7% | 0.90 [0.05, 15.07] |
| Yang 2023 | 2 | 64 | 1 | 58 | 5.7% | 1.84 [0.16, 20.83] |
| YU 2023 | 2 | 45 | 1 | 20 | 7.4% | 0.88 [0.08, 10.35] |
| Zou 2023 | 3 | 70 | 5 | 90 | 23.4% | 0.76 [0.18, 3.30] |
| **Total (95% CI)** | | **458** | | **420** | **100.0%** | **1.11 [0.59, 2.11]** |
| Total events | 22 | | 18 | | | |

Heterogeneity: Chi² = 2.22, df = 8 (P = 0.97); I² = 0%
Test for overall effect: Z = 0.33 (P = 0.74)

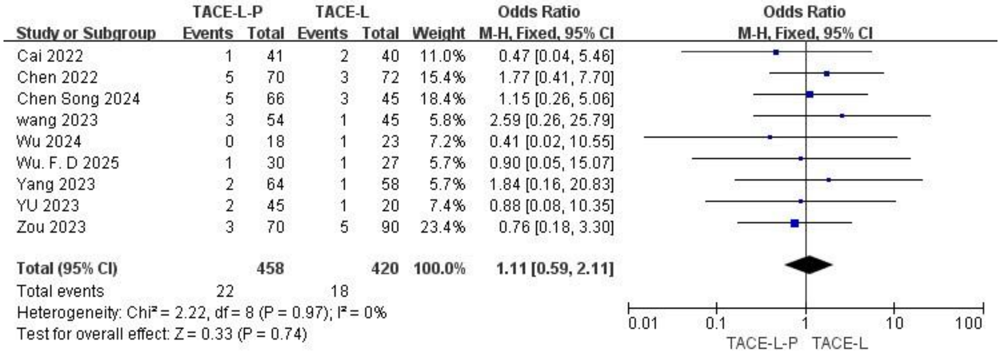


**Figure S21.**Subgroup analysis regarding Hypothyroidism

| Study or Subgroup | TACE-L-P Events | TACE-L-P Total | TACE-L Events | TACE-L Total | Weight | Odds Ratio M-H, Fixed, 95% CI |
|---|---|---|---|---|---|---|
| Chen 2022 | 4 | 70 | 1 | 72 | 10.6% | 4.30 [0.47, 39.49] |
| Chen Song 2024 | 3 | 66 | 2 | 45 | 25.9% | 1.02 [0.16, 6.39] |
| Ding 2024 | 1 | 19 | 0 | 16 | 5.7% | 2.68 [0.10, 70.31] |
| Sheng 2024 | 4 | 113 | 1 | 128 | 10.3% | 4.66 [0.51, 42.32] |
| Wu. F. D 2025 | 1 | 30 | 0 | 27 | 5.7% | 2.80 [0.11, 71.59] |
| Yang 2023 | 3 | 64 | 3 | 58 | 34.3% | 0.90 [0.17, 4.65] |
| YU 2023 | 2 | 45 | 0 | 20 | 7.4% | 2.36 [0.11, 51.34] |
| **Total (95% CI)** | | **407** | | **366** | **100.0%** | **2.00 [0.88, 4.53]** |
| Total events | 18 | | 7 | | | |

Heterogeneity: Chi² = 2.53, df = 6 (P = 0.87); I² = 0%
Test for overall effect: Z = 1.66 (P = 0.10)

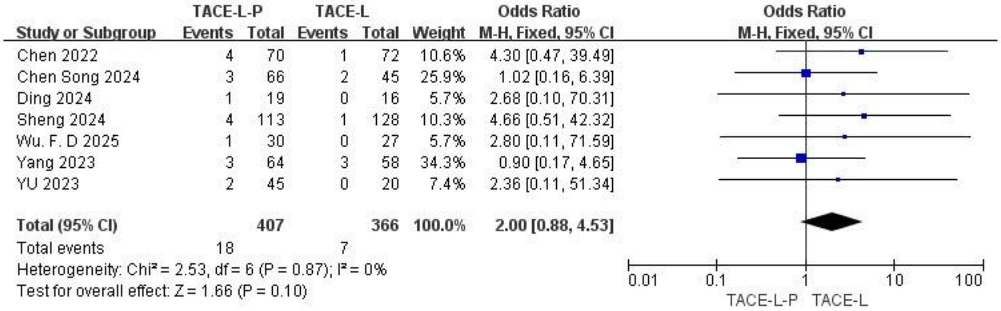


**Figure S22.**Subgroup analysis regarding Fever

| Study or Subgroup | TACE-L-P Events | TACE-L-P Total | TACE-L Events | TACE-L Total | Weight | Odds Ratio M-H, Fixed, 95% CI |
|---|---|---|---|---|---|---|
| Chen 2022 | 4 | 70 | 5 | 72 | 42.9% | 0.81 [0.21, 3.16] |
| Chen Song 2024 | 2 | 66 | 1 | 45 | 10.7% | 1.38 [0.12, 15.63] |
| Qu 2022 | 0 | 30 | 1 | 21 | 16.0% | 0.22 [0.01, 5.77] |
| Sheng 2024 | 0 | 113 | 1 | 128 | 12.9% | 0.37 [0.02, 9.28] |
| Wu. F. D 2025 | 3 | 30 | 2 | 27 | 17.5% | 1.39 [0.21, 9.01] |
| **Total (95% CI)** | | **309** | | **293** | **100.0%** | **0.82 [0.34, 1.99]** |
| Total events | 9 | | 10 | | | |

Heterogeneity: Chi² = 1.32, df = 4 (P = 0.86); I² = 0%
Test for overall effect: Z = 0.43 (P = 0.67)

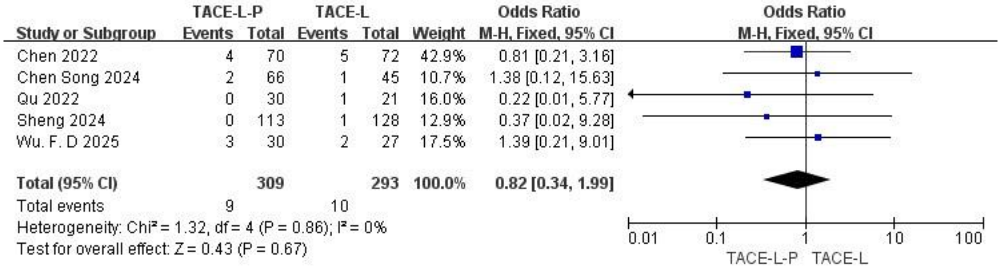


**Figure S23.** Forest plot of the meta-analysis for ORR (PD-1 Inhibitor Type subgroup analysis)

| Study or Subgroup | TACE-L-P Events | TACE-L-P Total | TACE-L Events | TACE-L Total | Weight | Odds Ratio M-H, Fixed, 95% CI |
|---|---|---|---|---|---|---|
| **5.2.1 Cam** | | | | | | |
| Lin 2023 | 19 | 45 | 15 | 50 | 5.7% | 1.71 [0.73, 3.97] |
| Xiang 2023 | 17 | 33 | 23 | 49 | 6.2% | 1.20 [0.50, 2.91] |
| **Subtotal (95% CI)** | | **78** | | **99** | **12.0%** | **1.44 [0.78, 2.65]** |
| Total events | 36 | | 38 | | | |
| Heterogeneity: Chi² = 0.32, df = 1 (P = 0.57); I² = 0% | | | | | | |
| Test for overall effect: Z = 1.18 (P = 0.24) | | | | | | |
| **5.2.2 Tis** | | | | | | |
| Chen Song 2024 | 53 | 66 | 24 | 45 | 3.9% | 3.57 [1.54, 8.29] |
| Jiang 2024 | 49 | 68 | 20 | 68 | 3.9% | 6.19 [2.94, 13.02] |
| Zhao. Y. H. 2024 | 68 | 103 | 31 | 66 | 8.9% | 2.19 [1.17, 4.13] |
| **Subtotal (95% CI)** | | **237** | | **179** | **16.7%** | **3.44 [2.28, 5.20]** |
| Total events | 170 | | 75 | | | |
| Heterogeneity: Chi² = 4.35, df = 2 (P = 0.11); I² = 54% | | | | | | |
| Test for overall effect: Z = 5.87 (P < 0.00001) | | | | | | |
| **5.2.3 Pem** | | | | | | |
| Chen 2022 | 33 | 70 | 20 | 72 | 7.3% | 2.32 [1.15, 4.66] |
| **Subtotal (95% CI)** | | **70** | | **72** | **7.3%** | **2.32 [1.15, 4.66]** |
| Total events | 33 | | 20 | | | |
| Heterogeneity: Not applicable | | | | | | |
| Test for overall effect: Z = 2.36 (P = 0.02) | | | | | | |
| **5.2.4 Tor** | | | | | | |
| Qu 2022 | 23 | 30 | 10 | 21 | 1.9% | 3.61 [1.08, 12.05] |
| **Subtotal (95% CI)** | | **30** | | **21** | **1.9%** | **3.61 [1.08, 12.05]** |
| Total events | 23 | | 10 | | | |
| Heterogeneity: Not applicable | | | | | | |
| Test for overall effect: Z = 2.09 (P = 0.04) | | | | | | |
| **5.2.5 Sin** | | | | | | |
| Wu. F. D 2025 | 21 | 30 | 15 | 27 | 3.3% | 1.87 [0.63, 5.55] |
| **Subtotal (95% CI)** | | **30** | | **27** | **3.3%** | **1.87 [0.63, 5.55]** |
| Total events | 21 | | 15 | | | |
| Heterogeneity: Not applicable | | | | | | |
| Test for overall effect: Z = 1.12 (P = 0.26) | | | | | | |
| **5.2.6 undistinguishable PD-1 types** | | | | | | |
| Cai 2022 | 23 | 41 | 13 | 40 | 4.0% | 2.65 [1.07, 6.56] |
| Ding 2024 | 6 | 19 | 5 | 16 | 2.6% | 1.02 [0.24, 4.26] |
| Guo 2022 | 51 | 75 | 48 | 91 | 9.7% | 1.90 [1.01, 3.60] |
| Huang 2022 | 32 | 44 | 26 | 50 | 4.6% | 2.46 [1.04, 5.85] |
| Sheng 2024 | 61 | 113 | 42 | 128 | 12.6% | 2.40 [1.42, 4.05] |
| wang 2023 | 38 | 54 | 22 | 45 | 4.9% | 2.48 [1.09, 5.67] |
| Wu 2024 | 3 | 18 | 3 | 23 | 1.5% | 1.33 [0.24, 7.56] |
| Yang 2023 | 37 | 64 | 24 | 58 | 7.4% | 1.94 [0.94, 3.99] |
| YU 2023 | 20 | 45 | 4 | 20 | 2.1% | 3.20 [0.92, 11.10] |
| Zhao 2022 | 20 | 23 | 15 | 32 | 1.1% | 7.56 [1.87, 30.58] |
| Zou 2023 | 27 | 70 | 22 | 90 | 8.2% | 1.94 [0.98, 3.83] |
| **Subtotal (95% CI)** | | **566** | | **593** | **58.9%** | **2.27 [1.77, 2.90]** |
| Total events | 318 | | 224 | | | |
| Heterogeneity: Chi² = 5.62, df = 10 (P = 0.85); I² = 0% | | | | | | |
| Test for overall effect: Z = 6.51 (P < 0.00001) | | | | | | |
| **Total (95% CI)** | | **1011** | | **991** | **100.0%** | **2.38 [1.98, 2.87]** |
| Total events | 601 | | 382 | | | |
| Heterogeneity: Chi² = 16.68, df = 18 (P = 0.55); I² = 0% | | | | | | |
| Test for overall effect: Z = 9.12 (P < 0.00001) | | | | | | |
| Test for subgroup differences: Chi² = 6.47, df = 5 (P = 0.26), I² = 22.8% | | | | | | |

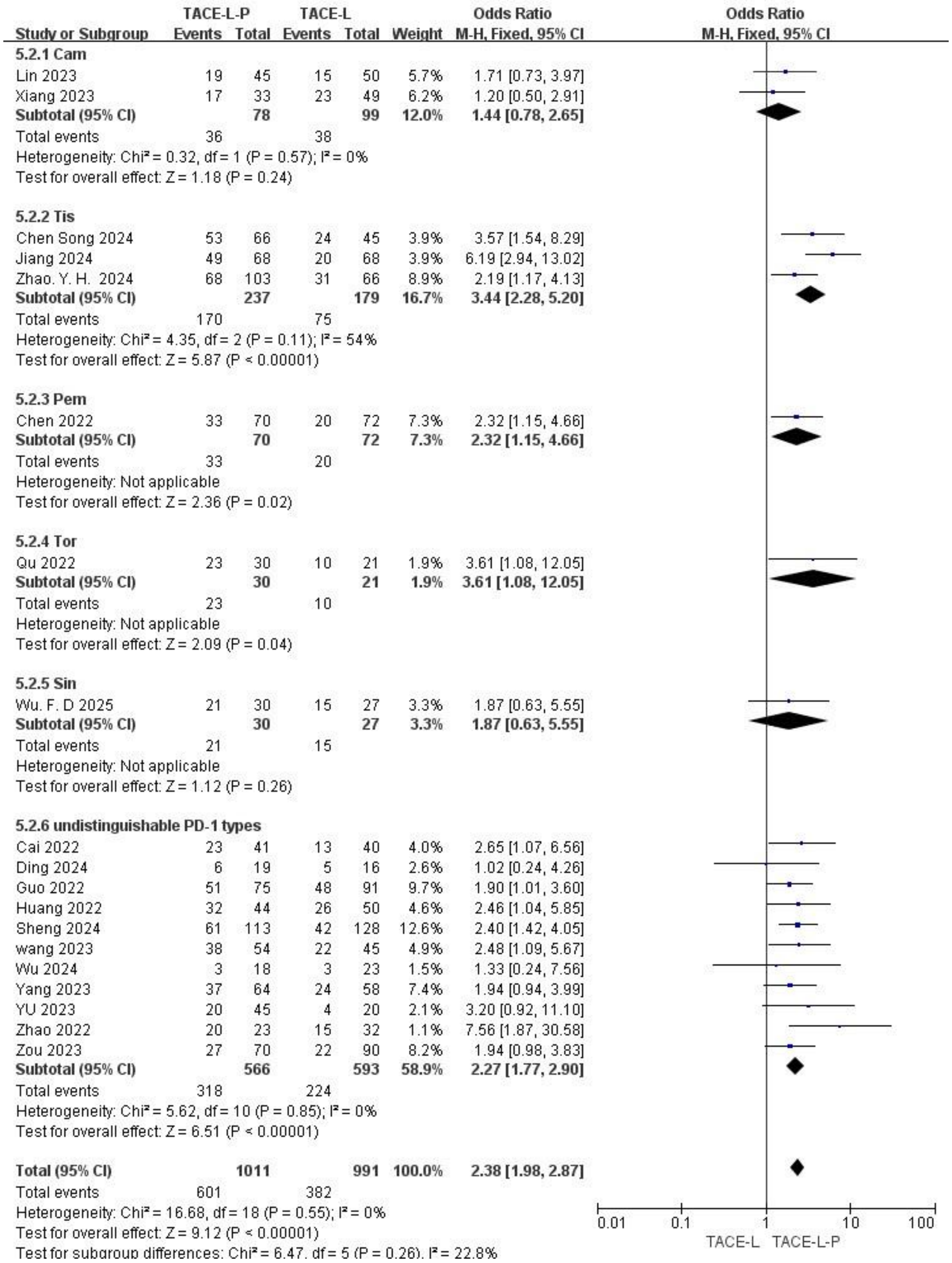


**Figure S24.** Forest plot of the meta-analysis for DCR (PD-1 Inhibitor Type subgroup analysis)

| Study or Subgroup | TACE-L-P Events | TACE-L-P Total | TACE-L Events | TACE-L Total | Weight | Odds Ratio M-H, Fixed, 95% CI |
|---|---|---|---|---|---|---|
| **5.1.1 Cam** | | | | | | |
| Lin 2023 | 31 | 45 | 25 | 50 | 9.5% | 2.21 [0.96, 5.13] |
| Xiang 2023 | 27 | 33 | 38 | 49 | 7.2% | 1.30 [0.43, 3.95] |
| **Subtotal (95% CI)** | | **78** | | **99** | **16.7%** | **1.82 [0.93, 3.56]** |
| Total events | 58 | | 63 | | | |
| Heterogeneity: Chi² = 0.56, df = 1 (P = 0.46); I² = 0% | | | | | | |
| Test for overall effect: Z = 1.76 (P = 0.08) | | | | | | |
| **5.1.2 Tis** | | | | | | |
| Chen Song 2024 | 62 | 66 | 36 | 66 | 2.8% | 12.92 [4.21, 39.64] |
| Jiang 2024 | 56 | 68 | 33 | 68 | 7.5% | 4.95 [2.26, 10.84] |
| Zhao. Y. H. 2024 | 98 | 103 | 61 | 66 | 4.7% | 1.61 [0.45, 5.78] |
| **Subtotal (95% CI)** | | **237** | | **200** | **15.0%** | **5.41 [3.12, 9.37]** |
| Total events | 216 | | 130 | | | |
| Heterogeneity: Chi² = 5.82, df = 2 (P = 0.05); I² = 66% | | | | | | |
| Test for overall effect: Z = 6.02 (P < 0.00001) | | | | | | |
| **5.1.3 Pem** | | | | | | |
| Chen 2022 | 49 | 70 | 38 | 72 | 14.6% | 2.09 [1.05, 4.16] |
| **Subtotal (95% CI)** | | **70** | | **72** | **14.6%** | **2.09 [1.05, 4.16]** |
| Total events | 49 | | 38 | | | |
| Heterogeneity: Not applicable | | | | | | |
| Test for overall effect: Z = 2.09 (P = 0.04) | | | | | | |
| **5.1.4 Tor** | | | | | | |
| Qu 2022 | 27 | 30 | 12 | 21 | 1.8% | 6.75 [1.55, 29.45] |
| **Subtotal (95% CI)** | | **30** | | **21** | **1.8%** | **6.75 [1.55, 29.45]** |
| Total events | 27 | | 12 | | | |
| Heterogeneity: Not applicable | | | | | | |
| Test for overall effect: Z = 2.54 (P = 0.01) | | | | | | |
| **5.1.5 Sin** | | | | | | |
| Wu. F. D 2025 | 27 | 30 | 17 | 27 | 2.3% | 5.29 [1.27, 22.03] |
| **Subtotal (95% CI)** | | **30** | | **27** | **2.3%** | **5.29 [1.27, 22.03]** |
| Total events | 27 | | 17 | | | |
| Heterogeneity: Not applicable | | | | | | |
| Test for overall effect: Z = 2.29 (P = 0.02) | | | | | | |
| **5.1.6 undistinguishable PD-1 types** | | | | | | |
| Cai 2022 | 35 | 41 | 25 | 40 | 4.8% | 3.50 [1.19, 10.27] |
| Ding 2024 | 15 | 19 | 15 | 16 | 4.4% | 0.25 [0.02, 2.51] |
| Guo 2022 | 62 | 75 | 68 | 91 | 13.8% | 1.61 [0.75, 3.46] |
| Huang 2022 | 38 | 44 | 31 | 50 | 5.1% | 3.88 [1.38, 10.91] |
| wang 2023 | 52 | 54 | 37 | 45 | 1.9% | 5.62 [1.13, 28.01] |
| Wu 2024 | 16 | 18 | 14 | 23 | 1.8% | 5.14 [0.95, 27.92] |
| Yang 2023 | 60 | 64 | 47 | 58 | 4.0% | 3.51 [1.05, 11.73] |
| YU 2023 | 42 | 45 | 13 | 20 | 1.6% | 7.54 [1.70, 33.41] |
| Zhao 2022 | 23 | 23 | 24 | 32 | 0.6% | 16.31 [0.89, 298.66] |
| Zou 2023 | 56 | 70 | 51 | 90 | 11.6% | 3.06 [1.49, 6.28] |
| **Subtotal (95% CI)** | | **453** | | **465** | **49.5%** | **3.03 [2.14, 4.30]** |
| Total events | 399 | | 325 | | | |
| Heterogeneity: Chi² = 11.15, df = 9 (P = 0.27); I² = 19% | | | | | | |
| Test for overall effect: Z = 6.21 (P < 0.00001) | | | | | | |
| **Total (95% CI)** | | **898** | | **884** | **100.0%** | **3.17 [2.49, 4.04]** |
| Total events | 776 | | 585 | | | |
| Heterogeneity: Chi² = 25.66, df = 17 (P = 0.08); I² = 34% | | | | | | |
| Test for overall effect: Z = 9.34 (P < 0.00001) | | | | | | |
| Test for subgroup differences: Chi² = 9.22, df = 5 (P = 0.10), I² = 45.8% | | | | | | |

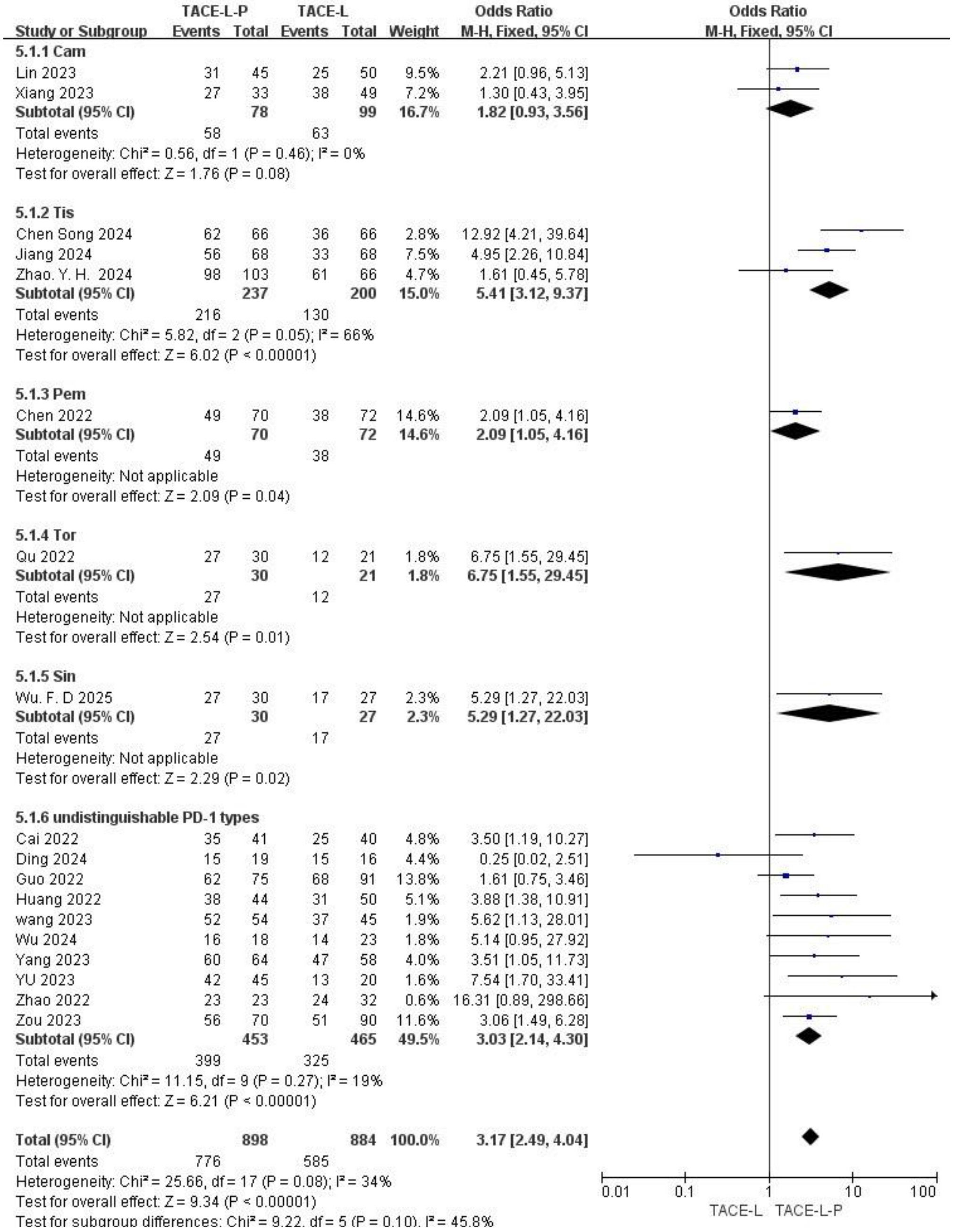


**Figure S25.** Forest plot of the meta-analysis for OS (PD-1 Inhibitor Type subgroup analysis)

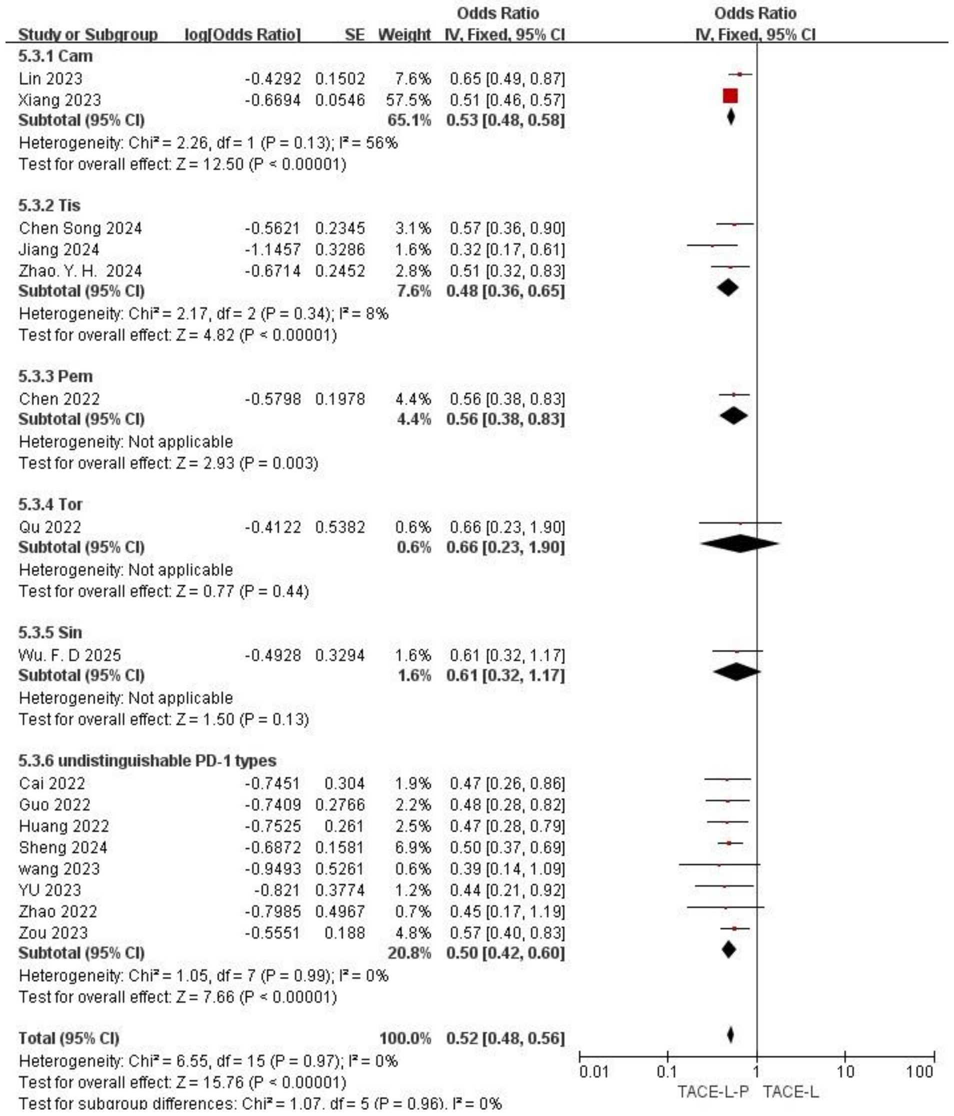


**Figure S26.** Forest plot of the meta-analysis for PFS (PD-1 Inhibitor Type subgroup analysis)

| Study or Subgroup | log[Hazard Ratio] | SE | Weight | Hazard Ratio IV, Fixed, 95% CI |
|---|---|---|---|---|
| **5.4.1 Cam** | | | | |
| Lin 2023 | -0.3481 | 0.1473 | 11.6% | 0.71 [0.53, 0.94] |
| Xiang 2023 | -0.2666 | 0.1229 | 16.7% | 0.77 [0.60, 0.97] |
| **Subtotal (95% CI)** | | | **28.3%** | **0.74 [0.62, 0.89]** |
| Heterogeneity: Chi² = 0.18, df = 1 (P = 0.67); I² = 0% | | | | |
| Test for overall effect: Z = 3.18 (P = 0.001) | | | | |
| **5.4.2 Tis** | | | | |
| Chen Song 2024 | -0.4943 | 0.2153 | 5.4% | 0.61 [0.40, 0.93] |
| Jiang 2024 | -0.9379 | 0.2342 | 4.6% | 0.39 [0.25, 0.62] |
| Zhao. Y. H. 2024 | -0.2666 | 0.1229 | 16.7% | 0.77 [0.60, 0.97] |
| **Subtotal (95% CI)** | | | **26.7%** | **0.65 [0.54, 0.79]** |
| Heterogeneity: Chi² = 6.56, df = 2 (P = 0.04); I² = 70% | | | | |
| Test for overall effect: Z = 4.41 (P < 0.0001) | | | | |
| **5.4.3 Pem** | | | | |
| Chen 2022 | -0.5108 | 0.2198 | 5.2% | 0.60 [0.39, 0.92] |
| **Subtotal (95% CI)** | | | **5.2%** | **0.60 [0.39, 0.92]** |
| Heterogeneity: Not applicable | | | | |
| Test for overall effect: Z = 2.32 (P = 0.02) | | | | |
| **5.4.4 Tor** | | | | |
| Qu 2022 | -0.6149 | 0.4131 | 1.5% | 0.54 [0.24, 1.22] |
| **Subtotal (95% CI)** | | | **1.5%** | **0.54 [0.24, 1.22]** |
| Heterogeneity: Not applicable | | | | |
| Test for overall effect: Z = 1.49 (P = 0.14) | | | | |
| **5.4.5 Sin** | | | | |
| Wu. F. D 2025 | -0.6687 | 0.3256 | 2.4% | 0.51 [0.27, 0.97] |
| **Subtotal (95% CI)** | | | **2.4%** | **0.51 [0.27, 0.97]** |
| Heterogeneity: Not applicable | | | | |
| Test for overall effect: Z = 2.05 (P = 0.04) | | | | |
| **5.4.6 undistinguishable PD-1 types** | | | | |
| Cai 2022 | -0.7934 | 0.273 | 3.4% | 0.45 [0.26, 0.77] |
| Ding 2024 | 0.1222 | 0.4698 | 1.1% | 1.13 [0.45, 2.84] |
| Guo 2022 | -0.4254 | 0.2864 | 3.1% | 0.65 [0.37, 1.15] |
| Huang 2022 | -0.6182 | 0.227 | 4.9% | 0.54 [0.35, 0.84] |
| Sheng 2024 | -0.6911 | 0.1918 | 6.8% | 0.50 [0.34, 0.73] |
| wang 2023 | -0.6714 | 0.3942 | 1.6% | 0.51 [0.24, 1.11] |
| Wu 2024 | -1.3863 | 0.3745 | 1.8% | 0.25 [0.12, 0.52] |
| Yang 2023 | -0.7744 | 0.1959 | 6.6% | 0.46 [0.31, 0.68] |
| YU 2023 | -0.755 | 0.3646 | 1.9% | 0.47 [0.23, 0.96] |
| Zhao 2022 | -0.5447 | 0.3196 | 2.5% | 0.58 [0.31, 1.09] |
| Zou 2023 | -0.7687 | 0.3256 | 2.4% | 0.46 [0.24, 0.88] |
| **Subtotal (95% CI)** | | | **36.0%** | **0.50 [0.43, 0.59]** |
| Heterogeneity: Chi² = 8.03, df = 10 (P = 0.63); I² = 0% | | | | |
| Test for overall effect: Z = 8.25 (P < 0.00001) | | | | |
| **Total (95% CI)** | | | **100.0%** | **0.61 [0.55, 0.67]** |
| Heterogeneity: Chi² = 25.30, df = 18 (P = 0.12); I² = 29% | | | | |
| Test for overall effect: Z = 9.95 (P < 0.00001) | | | | |
| Test for subgroup differences: Chi² = 10.54, df = 5 (P = 0.06), I² = 52.5% | | | | |

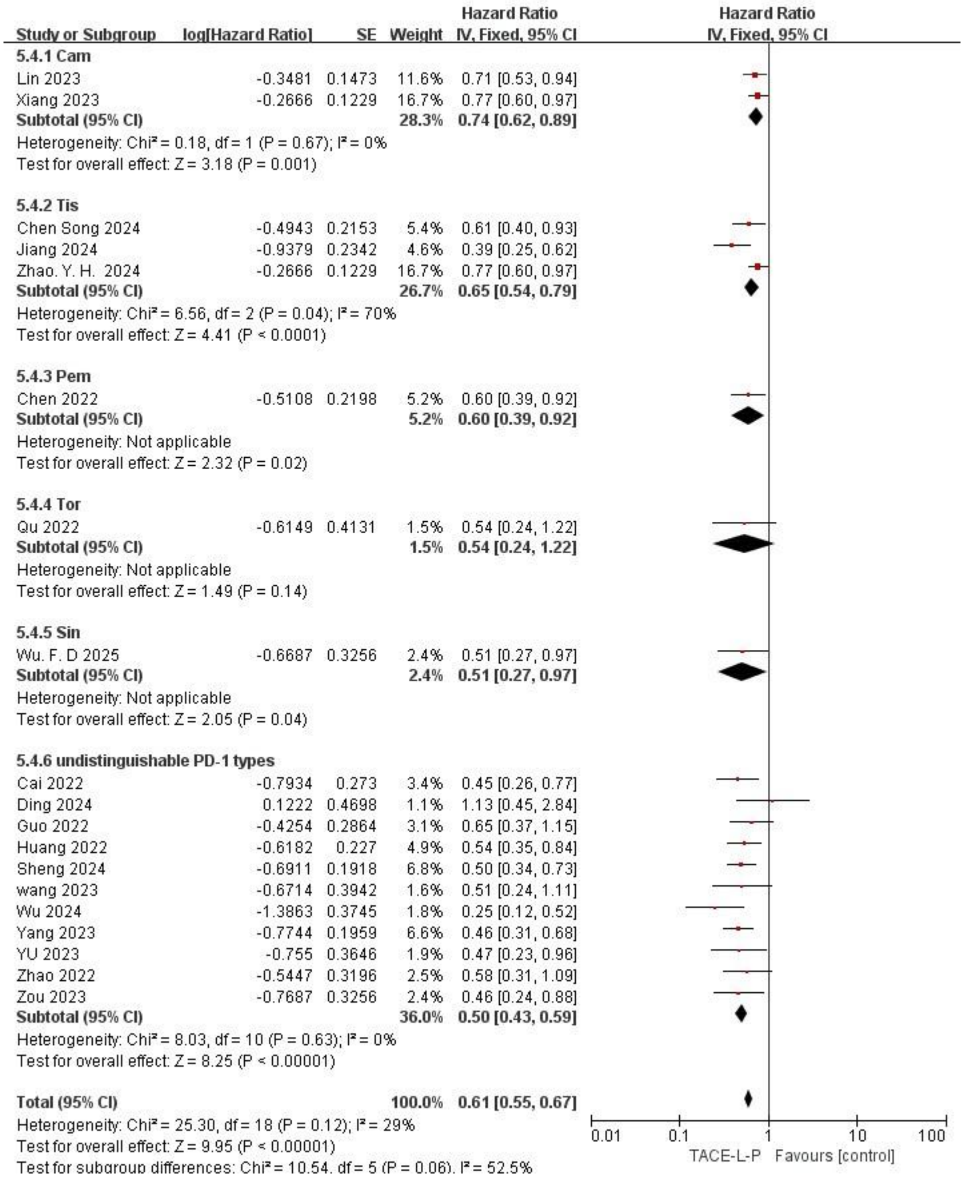

Supplement: Supplementary Material 2 — Supplementary Figures. [file DataSheet2.pdf]
